# Supplementary material for: Tunnel-structured IrOx unlocks catalytic efficiency in proton exchange membrane water electrolyzers
Source: Nat Commun. 2025 Aug 15;16:7608. doi: 10.1038/s41467-025-62861-0 (PMC12356887; doi:10.1038/s41467-025-62861-0)
Supplement: Supplementary file 1 — Supplementary Information [file 41467_2025_62861_MOESM1_ESM.pdf]

Supplementary Information

Tunnel-Structured IrO_x Unlocks Catalytic Efficiency in Proton Exchange Membrane Water Electrolyzers

Mingcheng Zhang,[1,†] Wei An,[1,†] Qianqian Liu,[1,2,†] Yuzhu Jiang,[1,†] Xiao Zhao,[3] Hui Chen,[1] Yongcun Zou,[1] Xiao Liang[1,*] and Xiaoxin Zou[1,*]

[1]State Key Laboratory of Inorganic Synthesis and Preparative Chemistry, College of Chemistry, Jilin University, Changchun 130012, China.

[2]School of Materials Science and Engineering, Xi'an University of Science and Technology, Xi'an 710054, China.

[3]Key Laboratory of Automobile Materials of MOE, School of Materials Science and Engineering, Jilin University, Changchun 130012, China.

*E-mail: liangxiao@jlu.edu.cn (X.L.); xxzou@jlu.edu.cn (X.Z.)

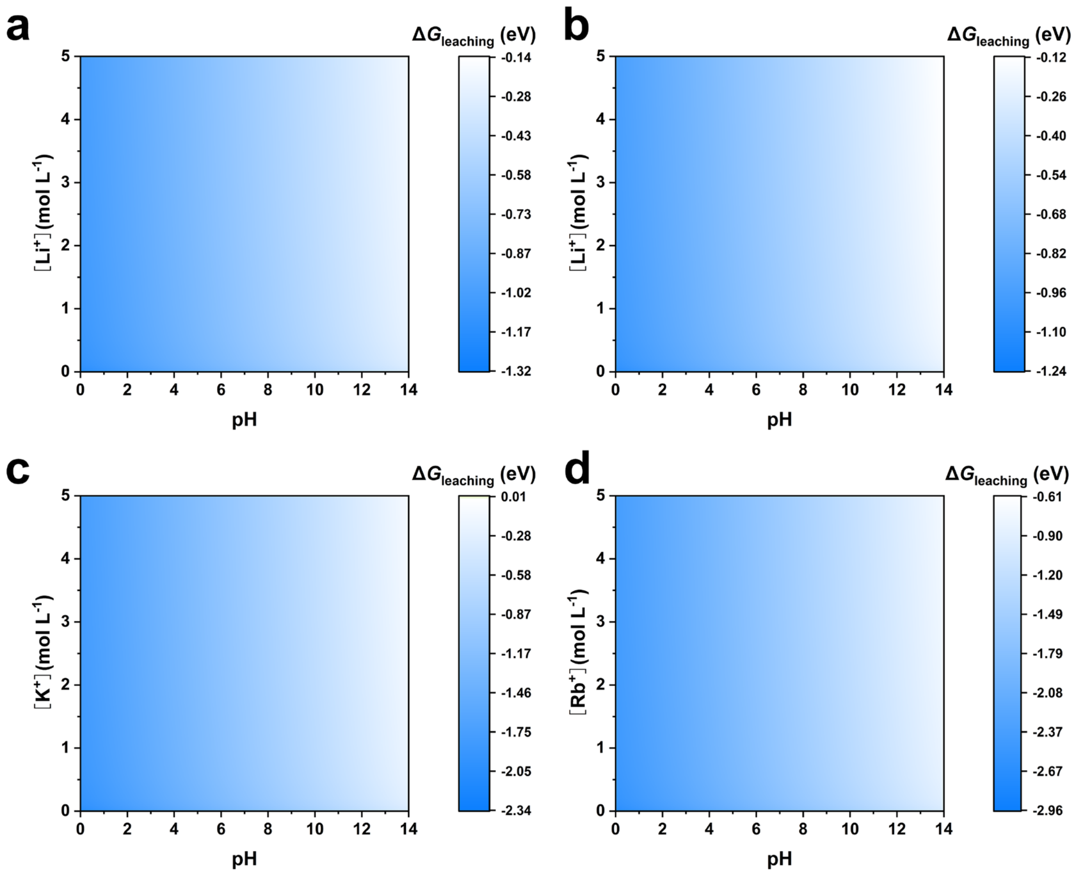

Supplementary Fig. 1 The $\Delta G_{\mathrm{leaching}}$ for A^+/H^+ exchange (A = Alkali metal) of (a) 1×2 (b) 1×2' (c) 2×3 and (d) 3×3 tunnel-structured iridates at different pH. Source data are provided as a Source Data file.

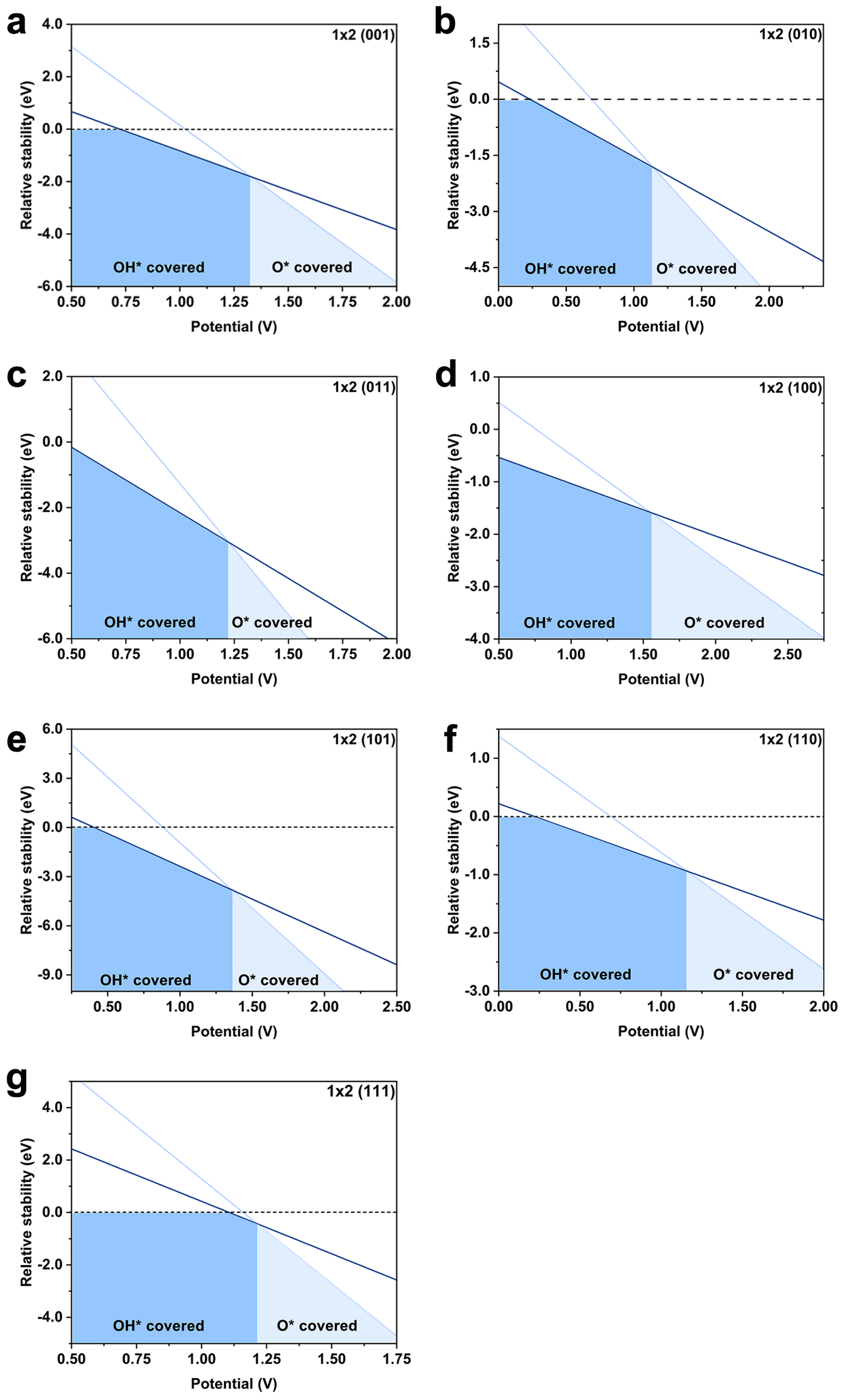

Supplementary Fig. 2 Surface Pourbaix diagrams for the 1×2 tunnel iridium oxides on different crystal planes: (a) (001), (b) (010), (c) (011), (d) (100), (e) (101), (f) (110), and (g) (111). Source data are provided as a Source Data file.

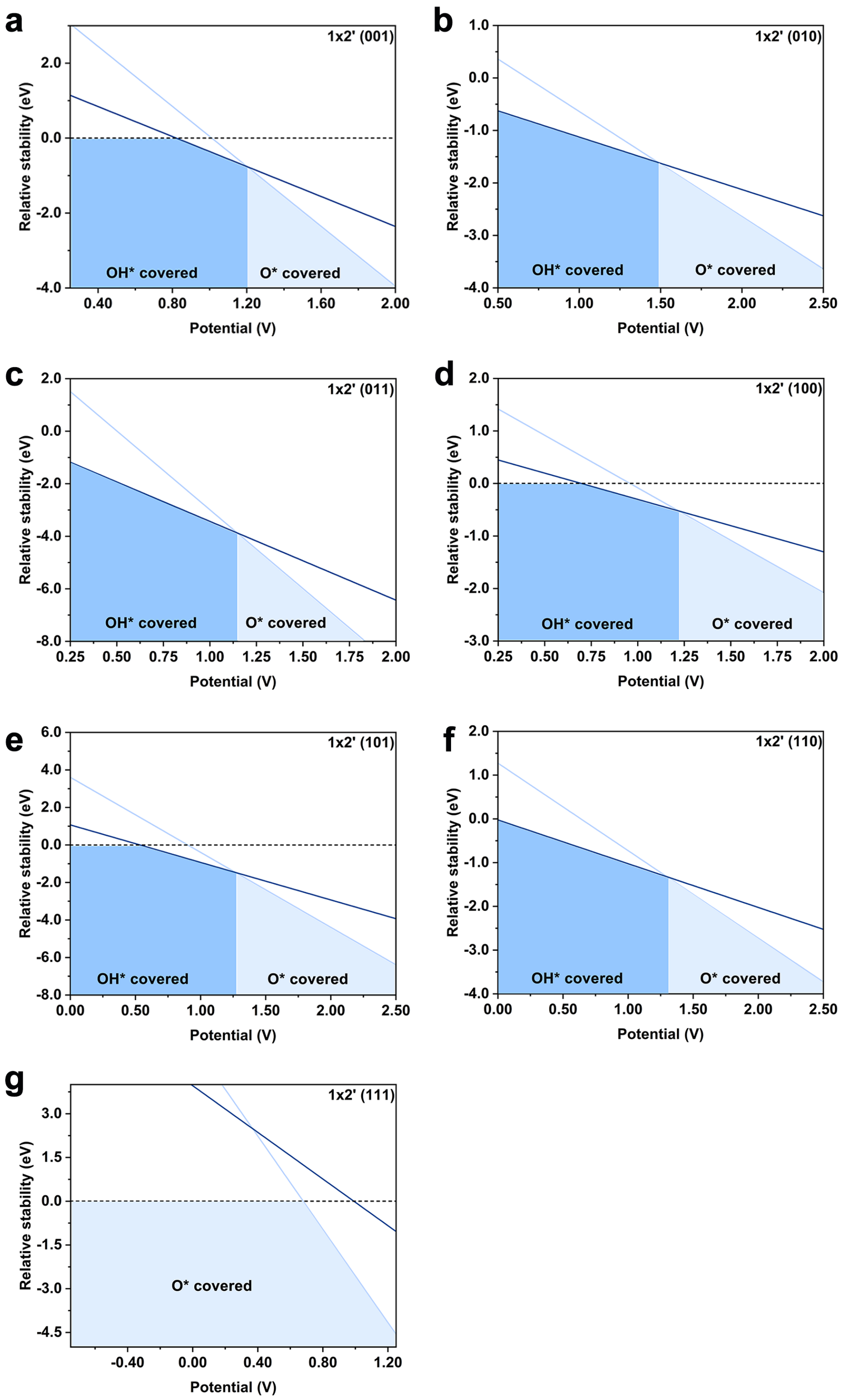

Supplementary Fig. 3 Surface Pourbaix diagrams for the 1×2' tunnel iridium oxides on different crystal planes: (a) (001), (b) (010), (c) (011), (d) (100), (e) (101), (f) (110), and (g) (111). Source data are provided as a Source Data file.

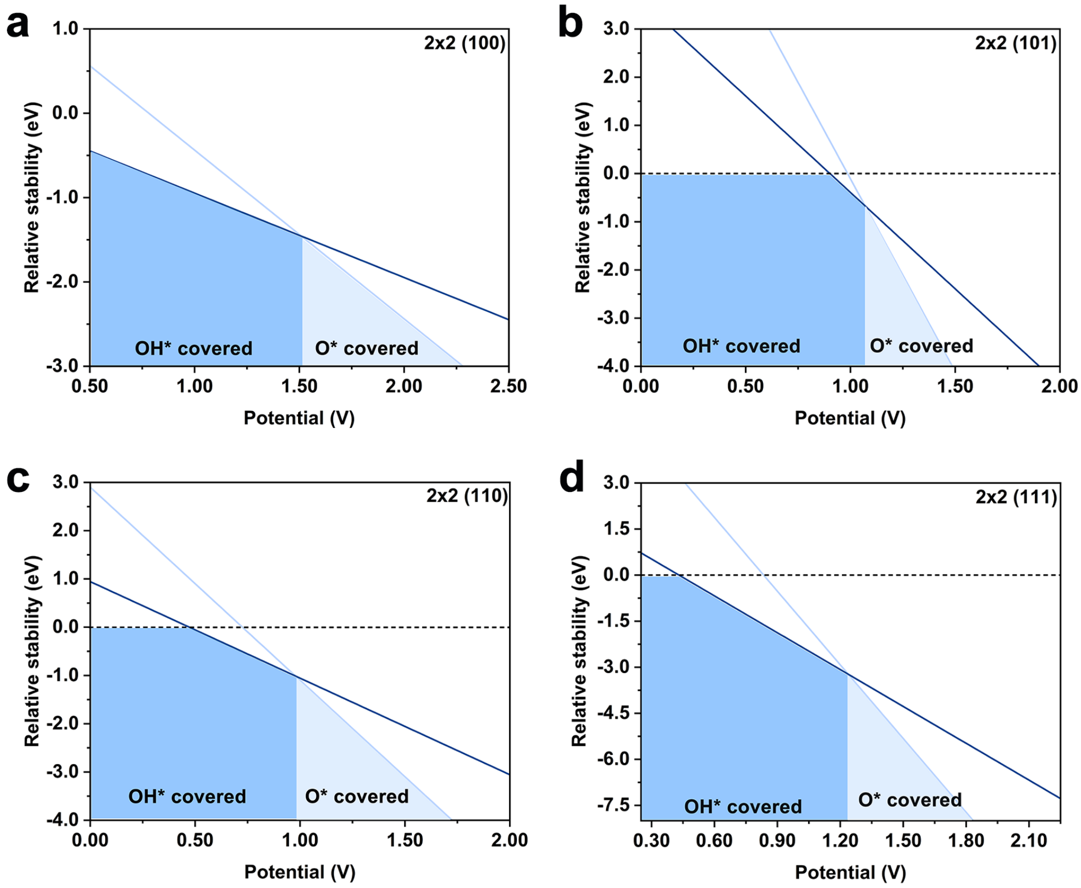

Supplementary Fig. 4 Surface Pourbaix diagrams for the 2×2 tunnel iridium oxides on different crystal planes: (a) (100), (b) (101), (c) (110), and (d) (111). Source data are provided as a Source Data file.

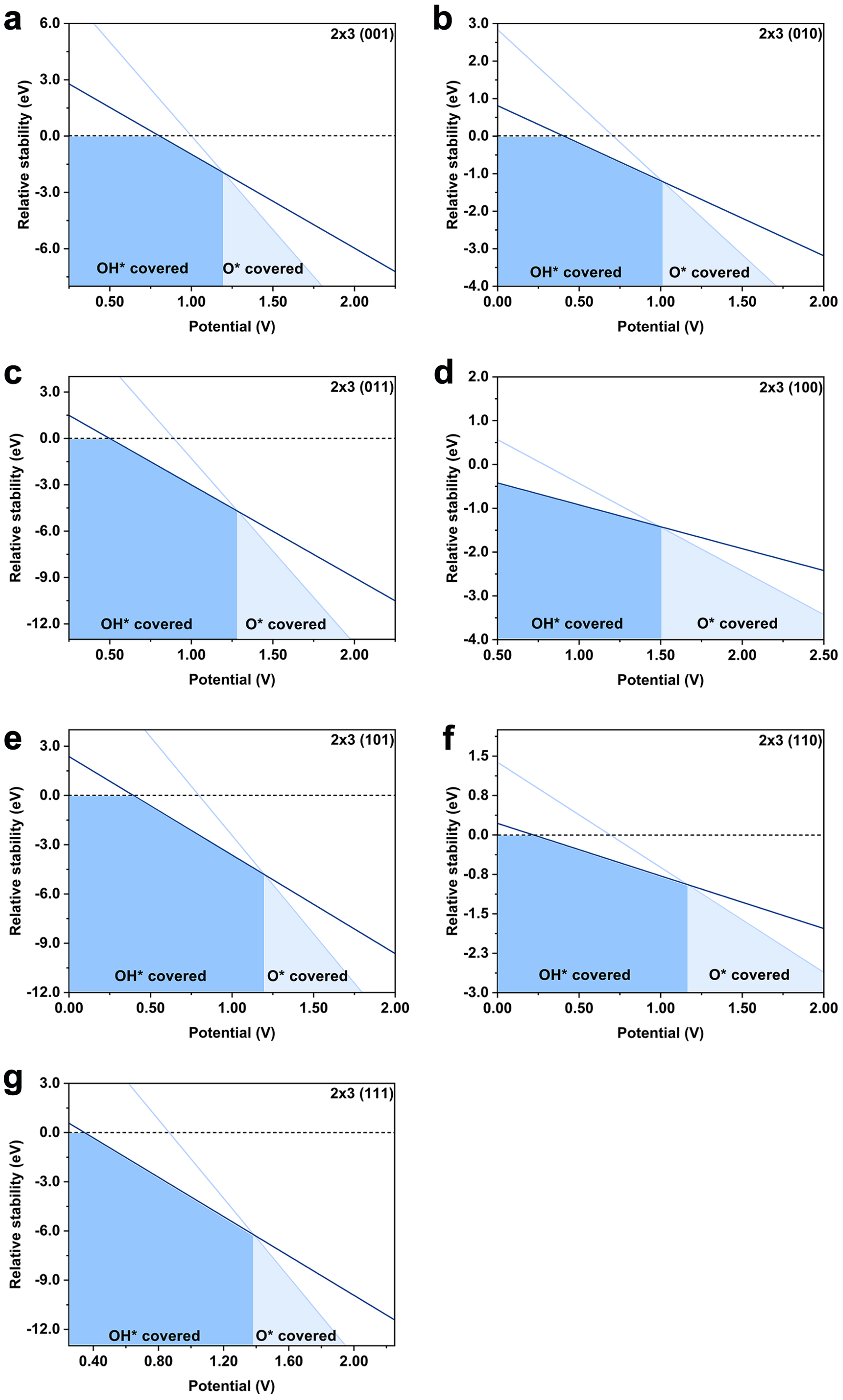

Supplementary Fig. 5 Surface Pourbaix diagrams for the 2×3 tunnel iridium oxides on different crystal planes: (a) (001), (b) (010), (c) (011), (d) (100), (e) (101), (f) (110), and (g) (111). Source data are provided as a Source Data file.

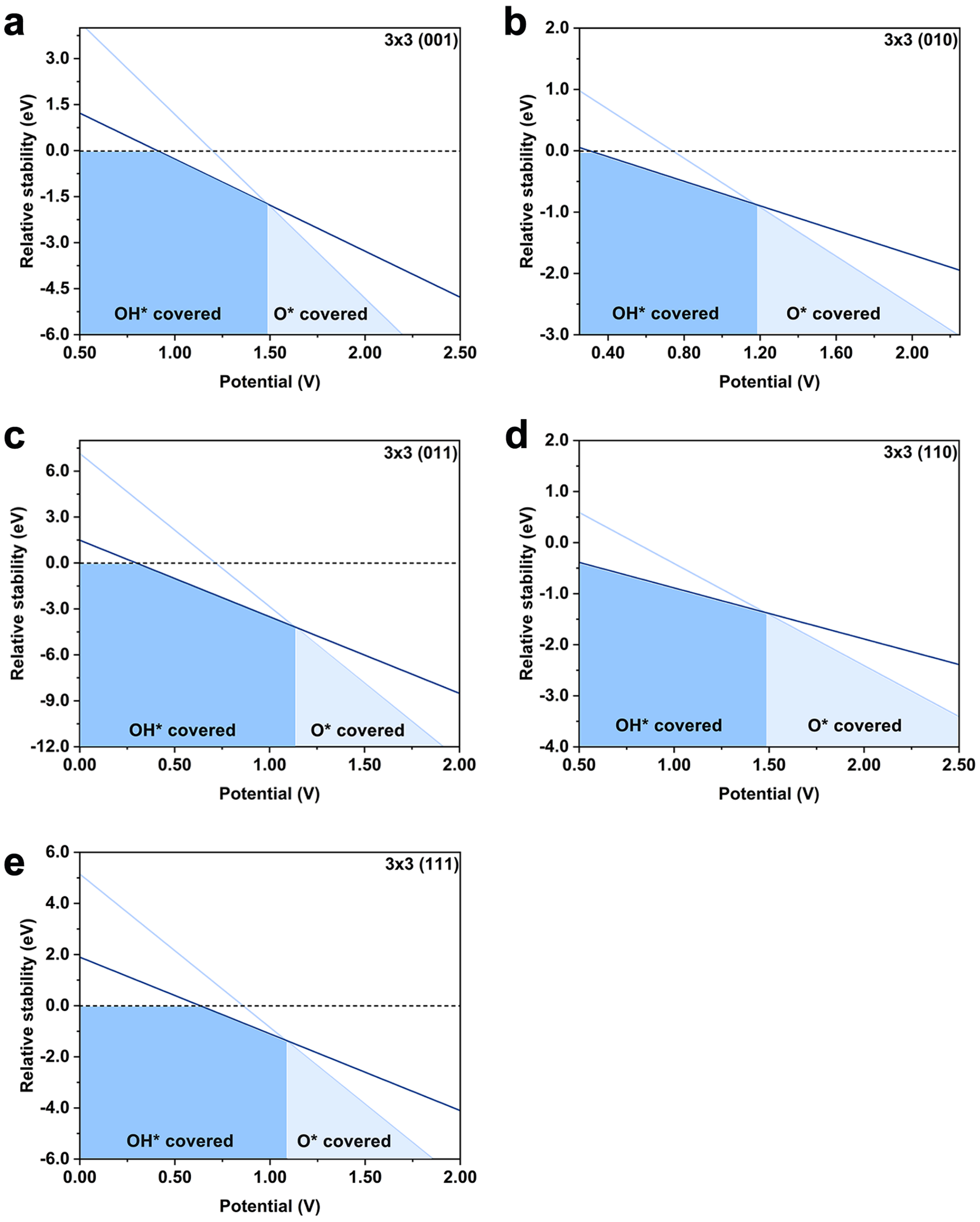

Supplementary Fig. 6 Surface Pourbaix diagrams for the 3×3 tunnel iridium oxides on different crystal planes: (a) (001), (b) (010), (c) (011), (d) (110), and (e) (111). Source data are provided as a Source Data file.

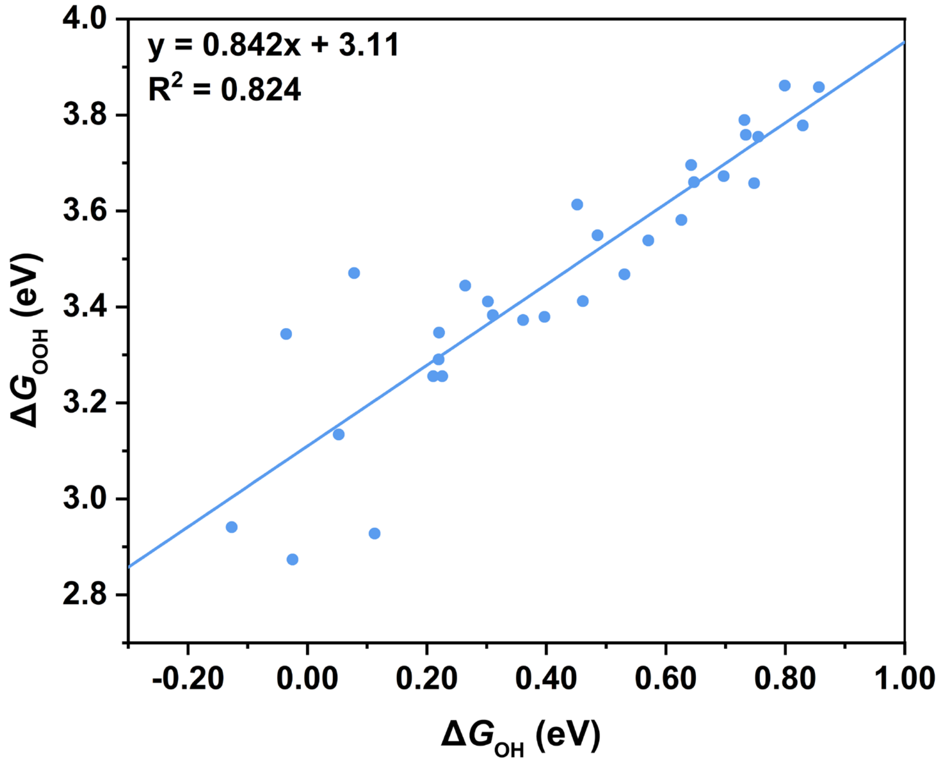

Supplementary Fig. 7 Scaling relationship between ΔG_{OH} and ΔG_{OOH} across five tunnel iridium oxides. Source data are provided as a Source Data file.

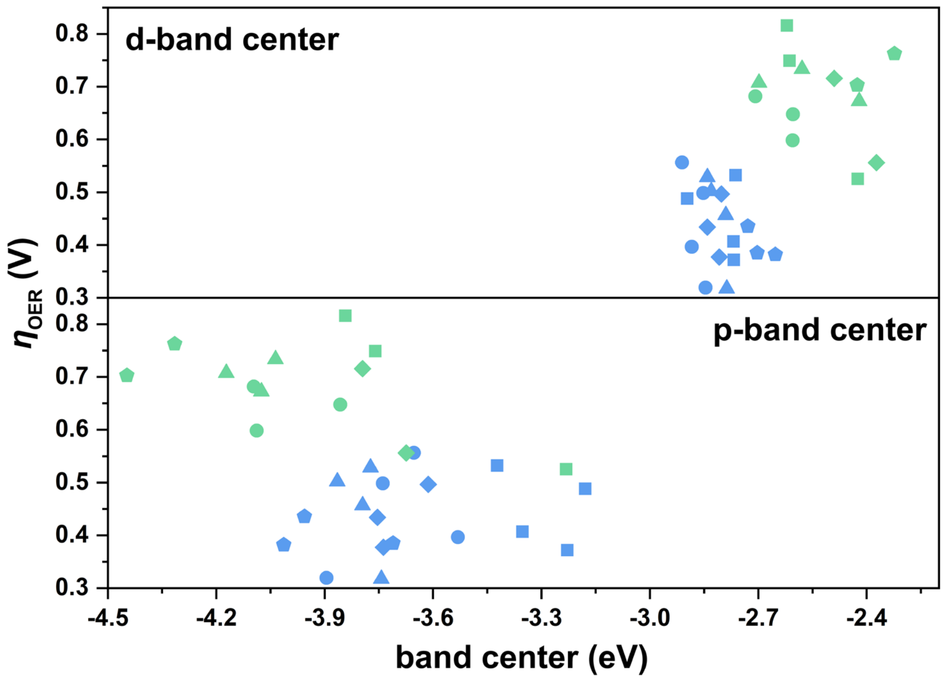

Supplementary Fig. 8 Correlation between d-band/p-band center and OER activity across five tunnel iridium oxides. Source data are provided as a Source Data file.

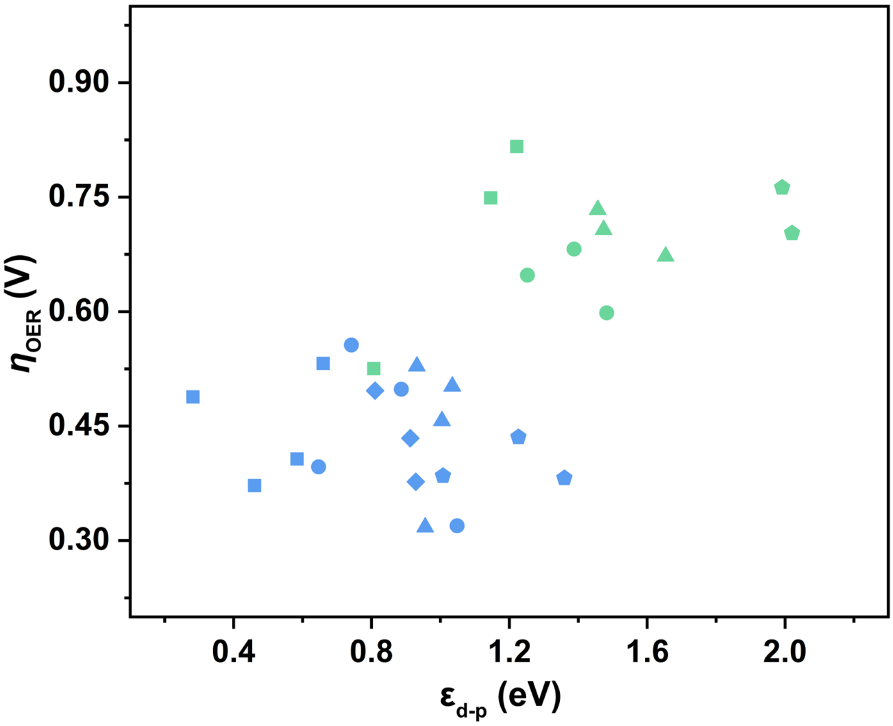

Supplementary Fig. 9 Correlation between charge transfer energies (ε_{d-p}) and OER activity (η_{OER}) across five tunnel iridium oxides. Source data are provided as a Source Data file.

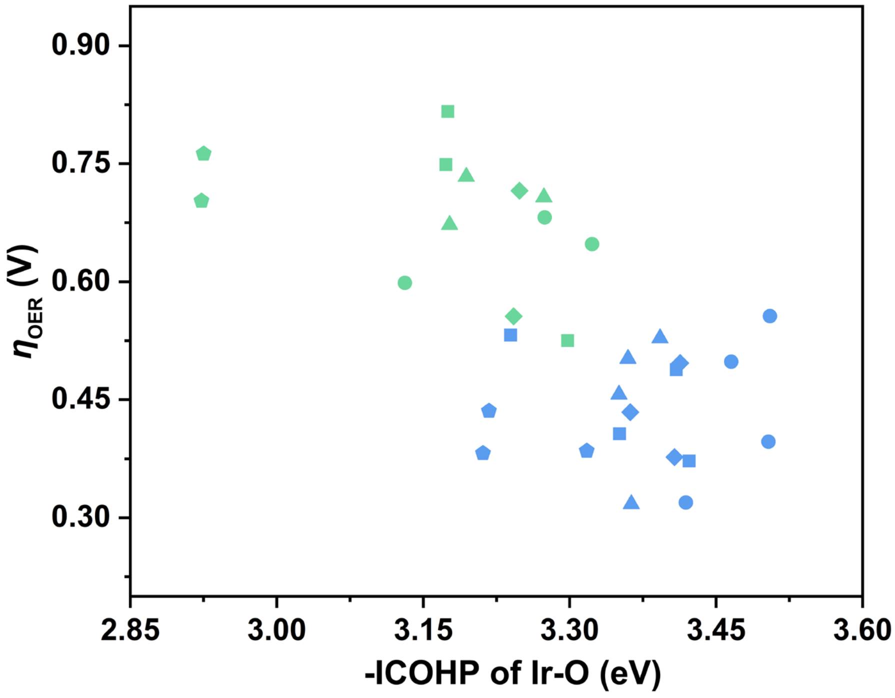

Supplementary Fig. 10 Correlation between integrated Crystal Orbital Hamilton Populations (–ICOHP) of Ir-O and OER activity (η_{OER}) across five tunnel iridium oxides. Source data are provided as a Source Data file.

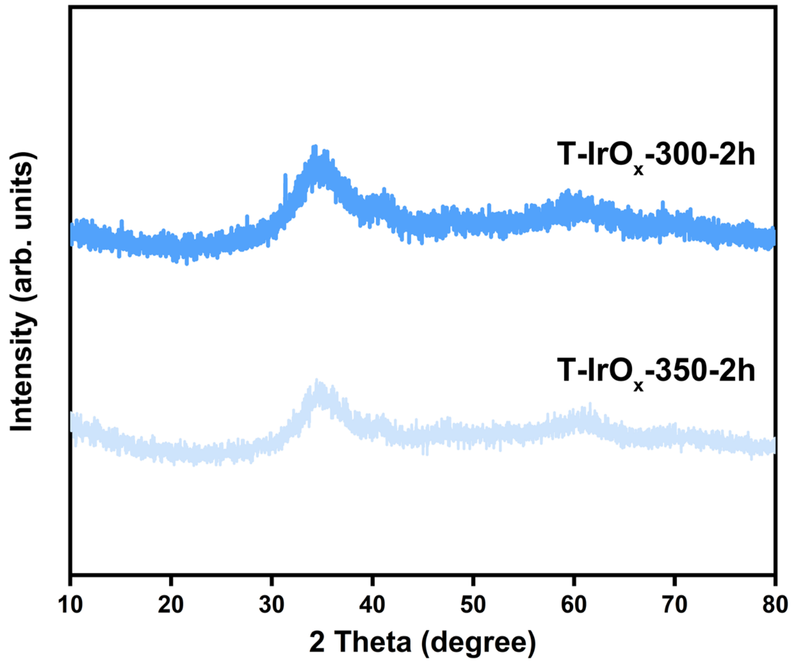

Supplementary Fig. 11 XRD patterns of T-IrO$_x$ synthesized at 300 and 350°C. Source data are provided as a Source Data file.

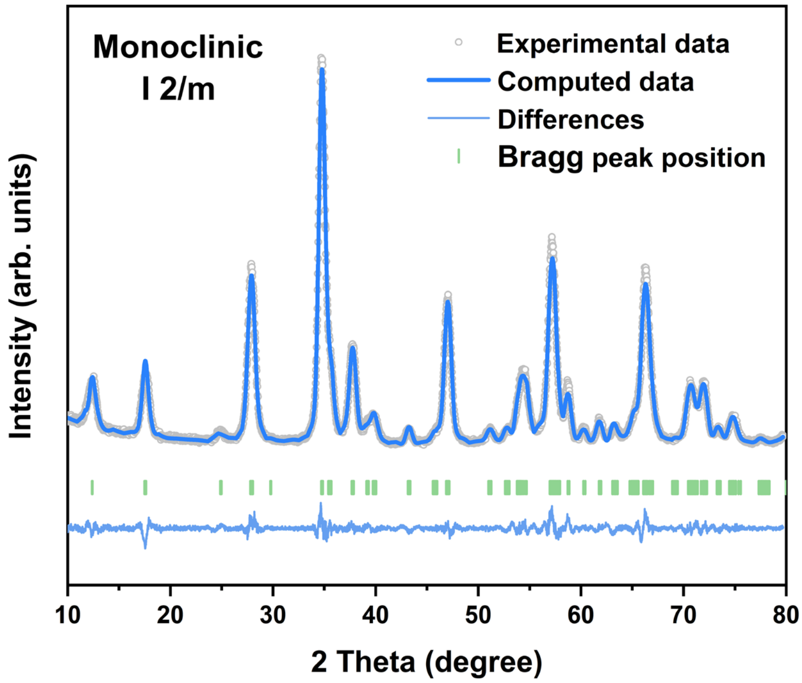

Supplementary Fig. 12 XRD pattern and Rietveld refinement of T-IrO$_x$-400. The refined crystal structure corresponds to a monoclinic system with space group I2/m, consistent with the standard PDF card (PDF# 85-2185). Source data are provided as a Source Data file.

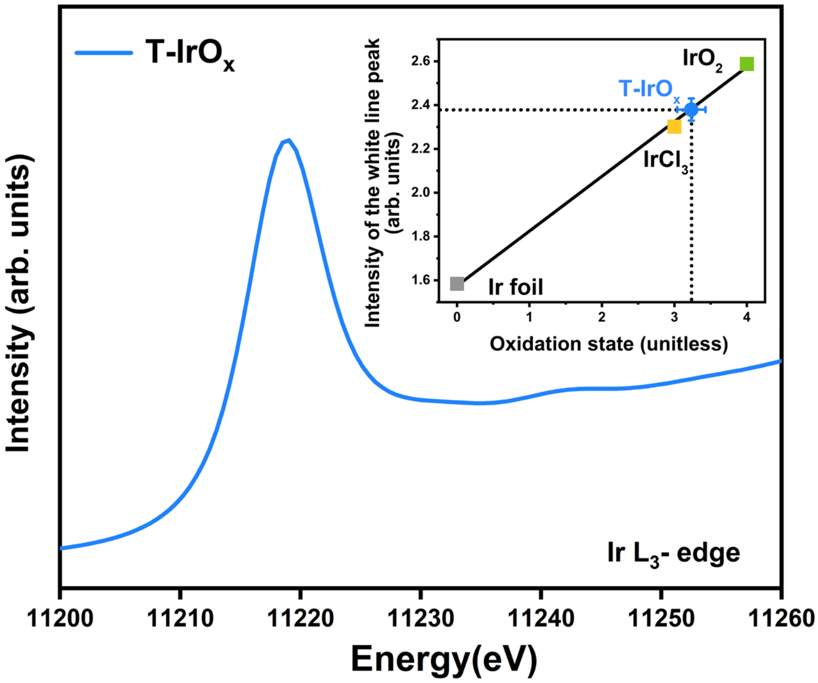

Supplementary Fig. 13 Normalized XANES spectra at Ir L_3-edge for T-IrO_x, IrO_2, $IrCl_3$, and Ir foil. The inset shows the fitted iridium oxidation states derived from the L_3-edge white line intensity. Source data are provided as a Source Data file.

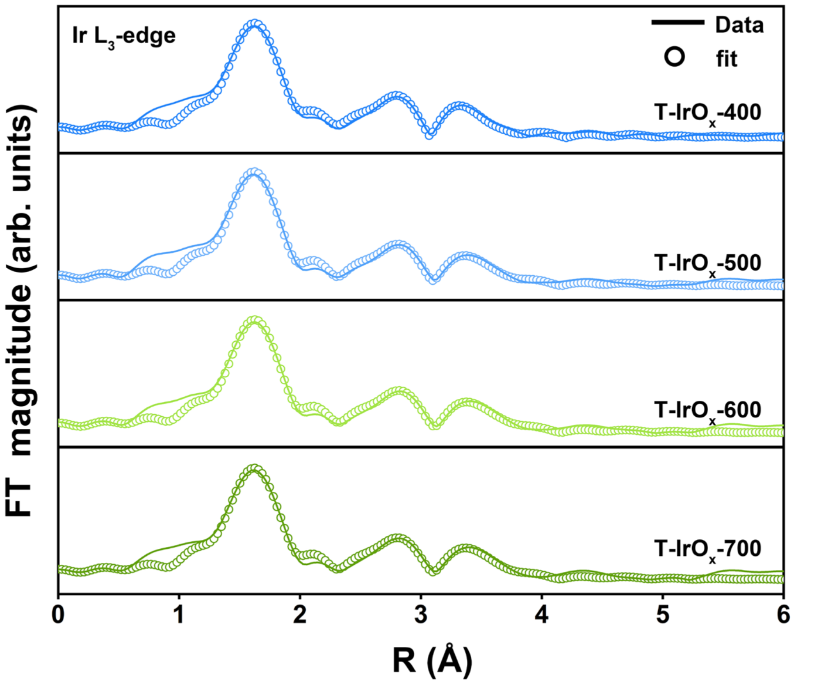

Supplementary Fig. 14 Fourier-transformed Ir L_3-edge EXAFS spectra of T-IrO_x-400, T-IrO_x-500, T-IrO_x-600 and T-IrO_x-700. Source data are provided as a Source Data file.

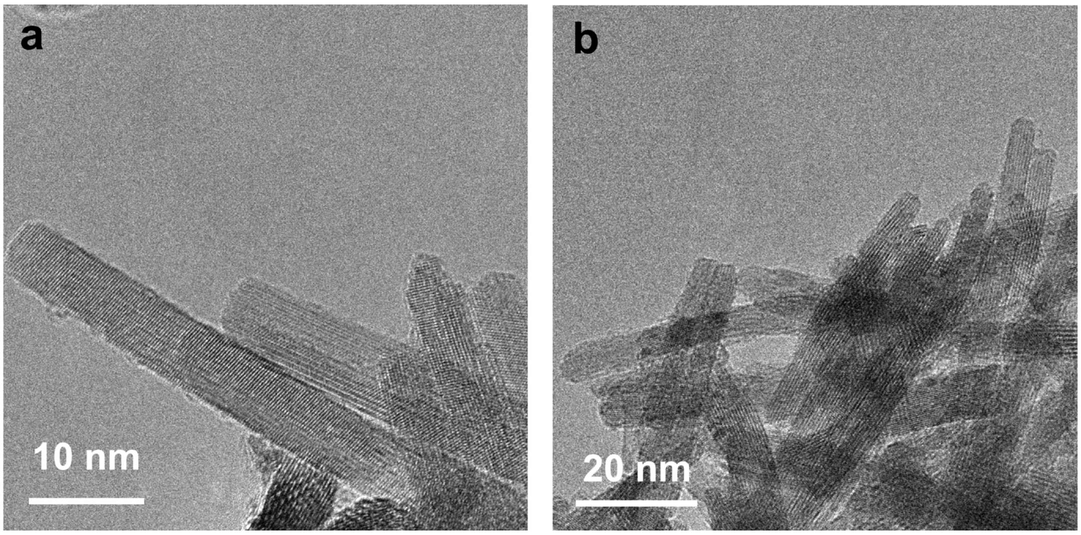

Supplementary Fig. 15 TEM images of T-IrO_x-400 nanorod tips.

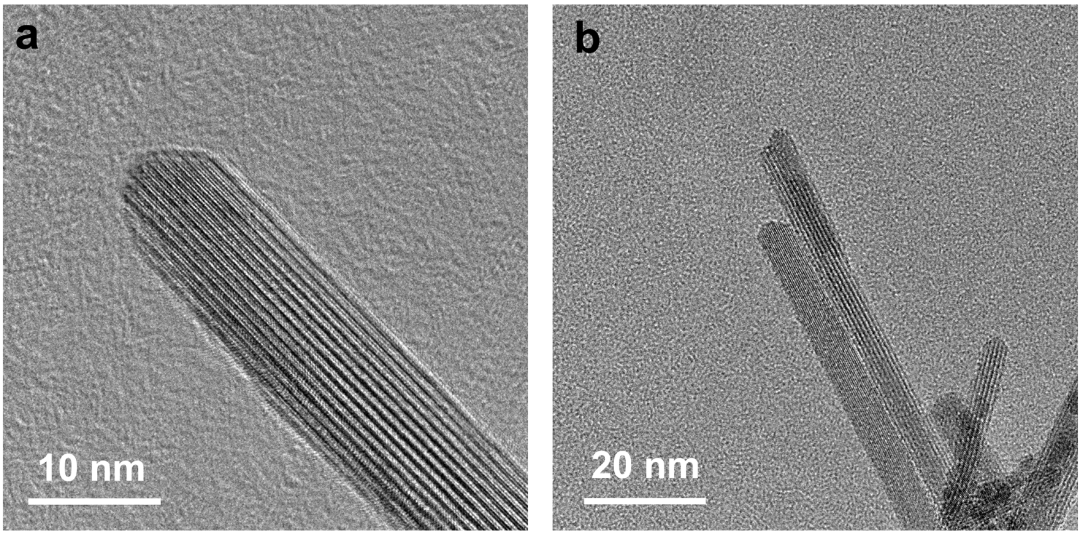

Supplementary Fig. 16 TEM images of T-IrO_x-500 nanorod tips.

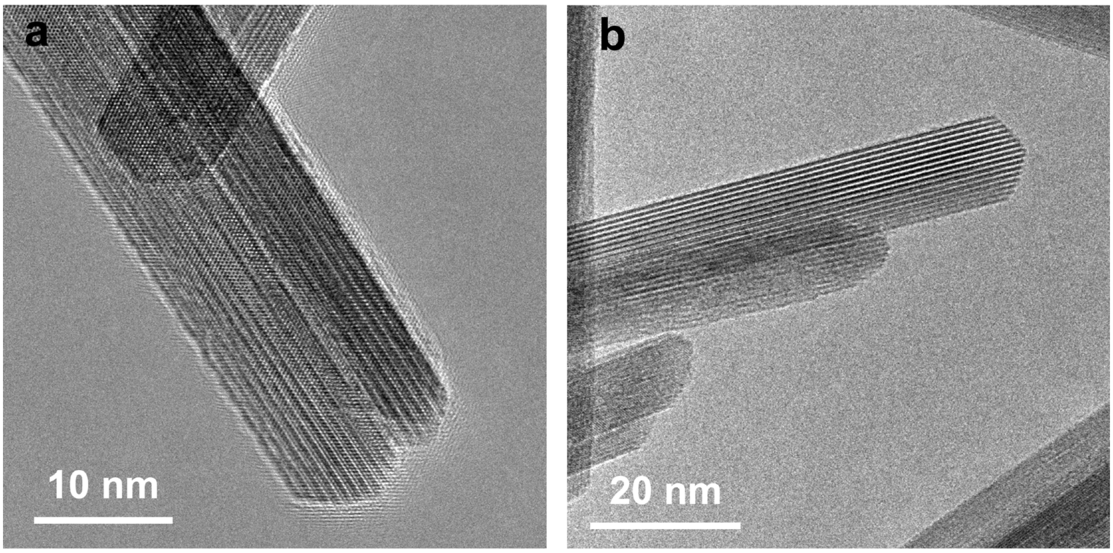

Supplementary Fig. 17 TEM images of T-IrO_x-600 nanorod tips.

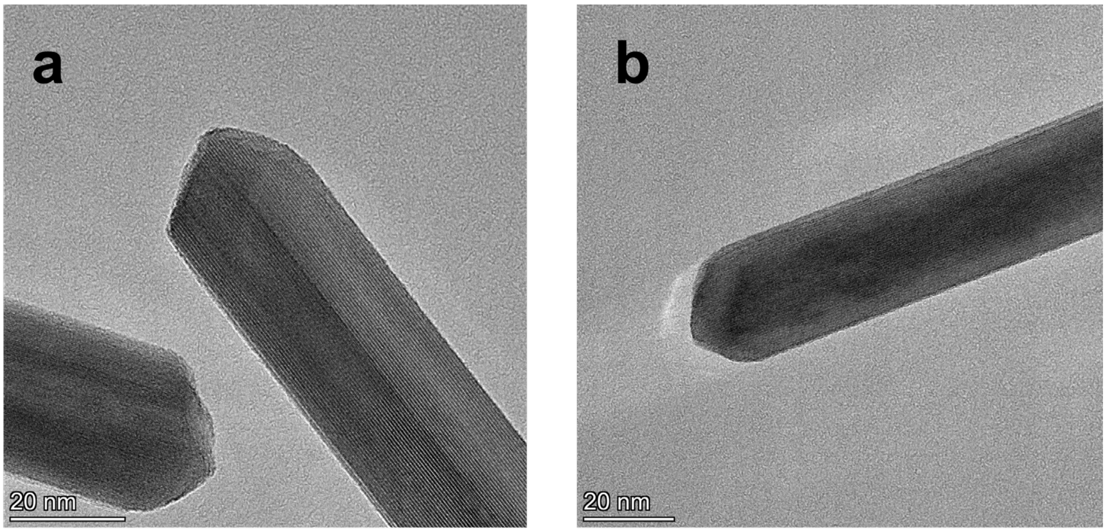

Supplementary Fig. 18 TEM images of T-IrO_x-700 nanorod tips.

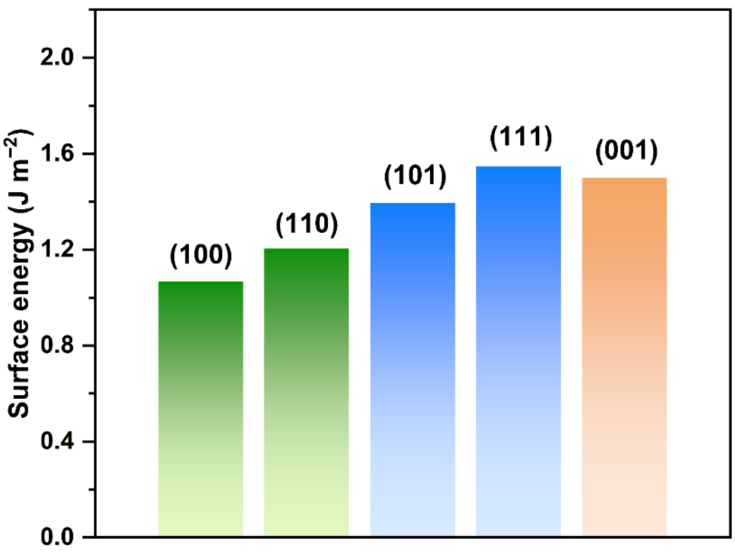

Supplementary Fig. 19 Surface energies of different crystal planes for the T-IrO_x. Source data are provided as a Source Data file.

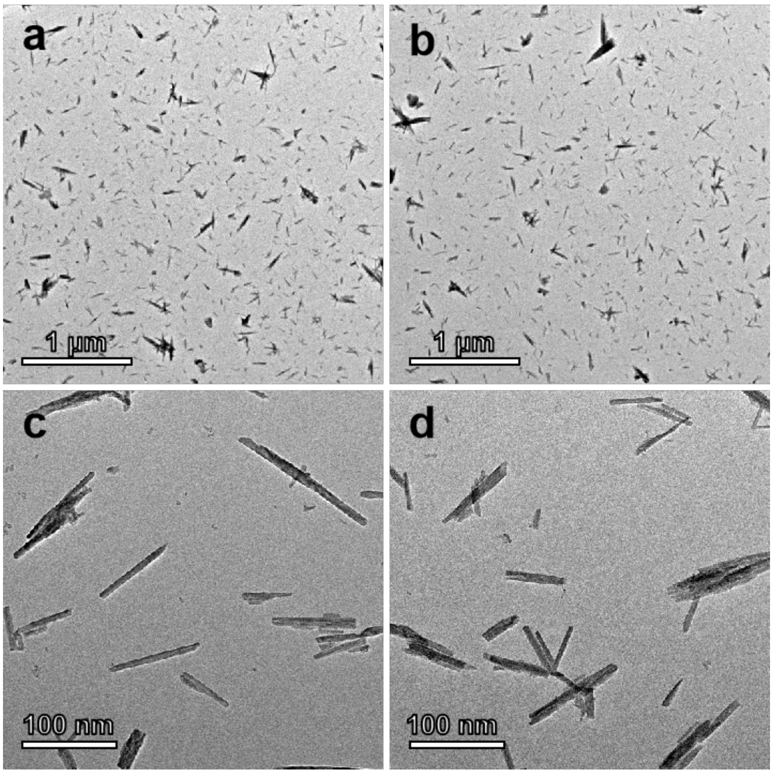

Supplementary Fig. 20 TEM images of T-IrO_x-400.

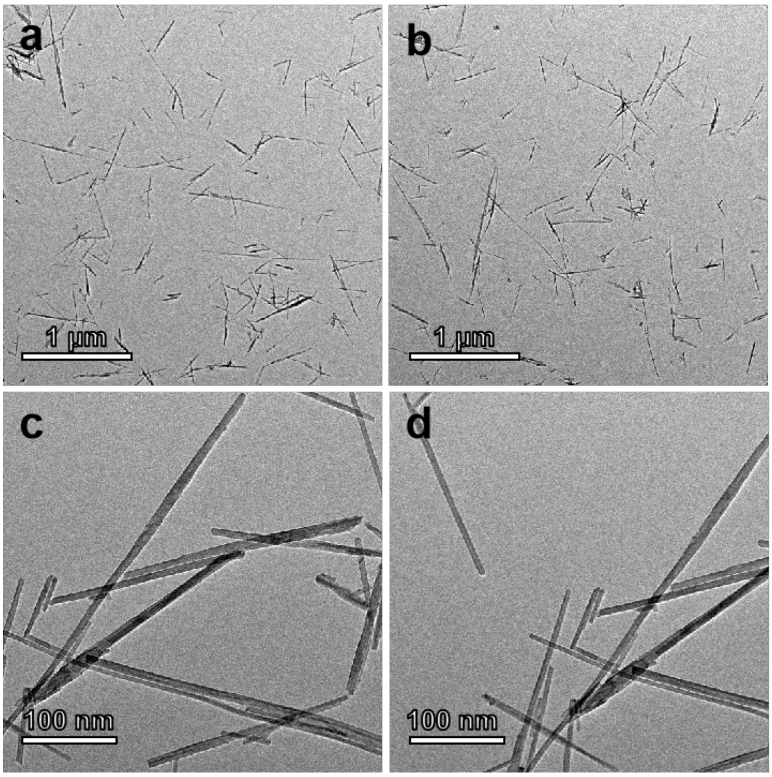

Supplementary Fig. 21 TEM images of T-IrO_x-500.

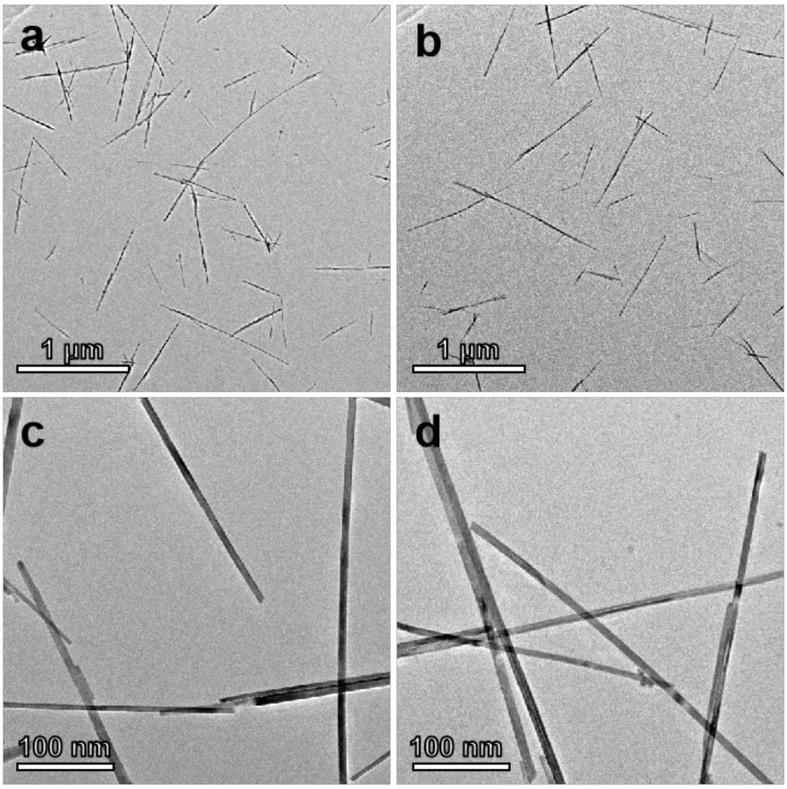

Supplementary Fig. 22 TEM images of T-IrO_x-600.

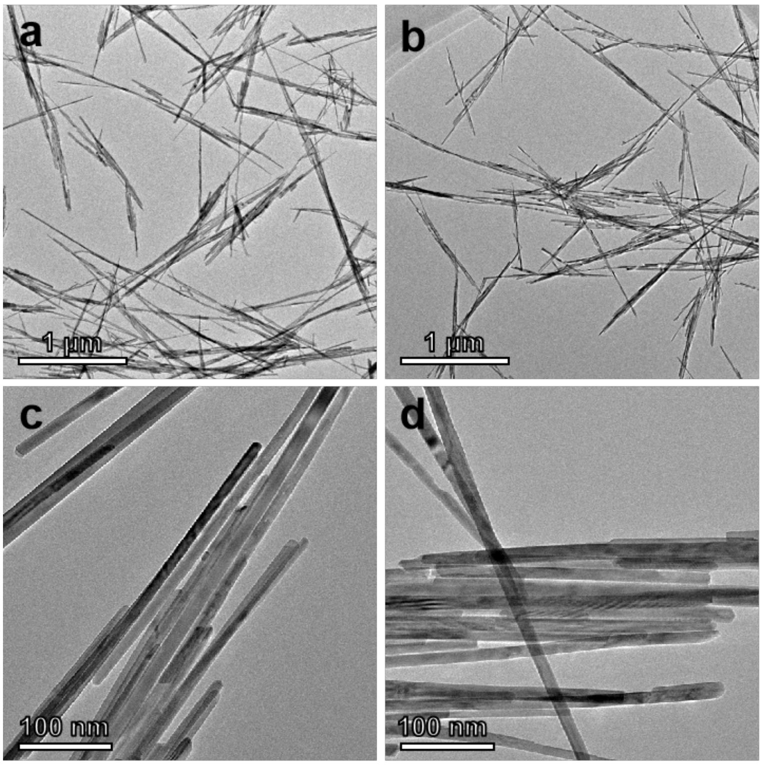

Supplementary Fig. 23 TEM images of T-IrO_x-700.

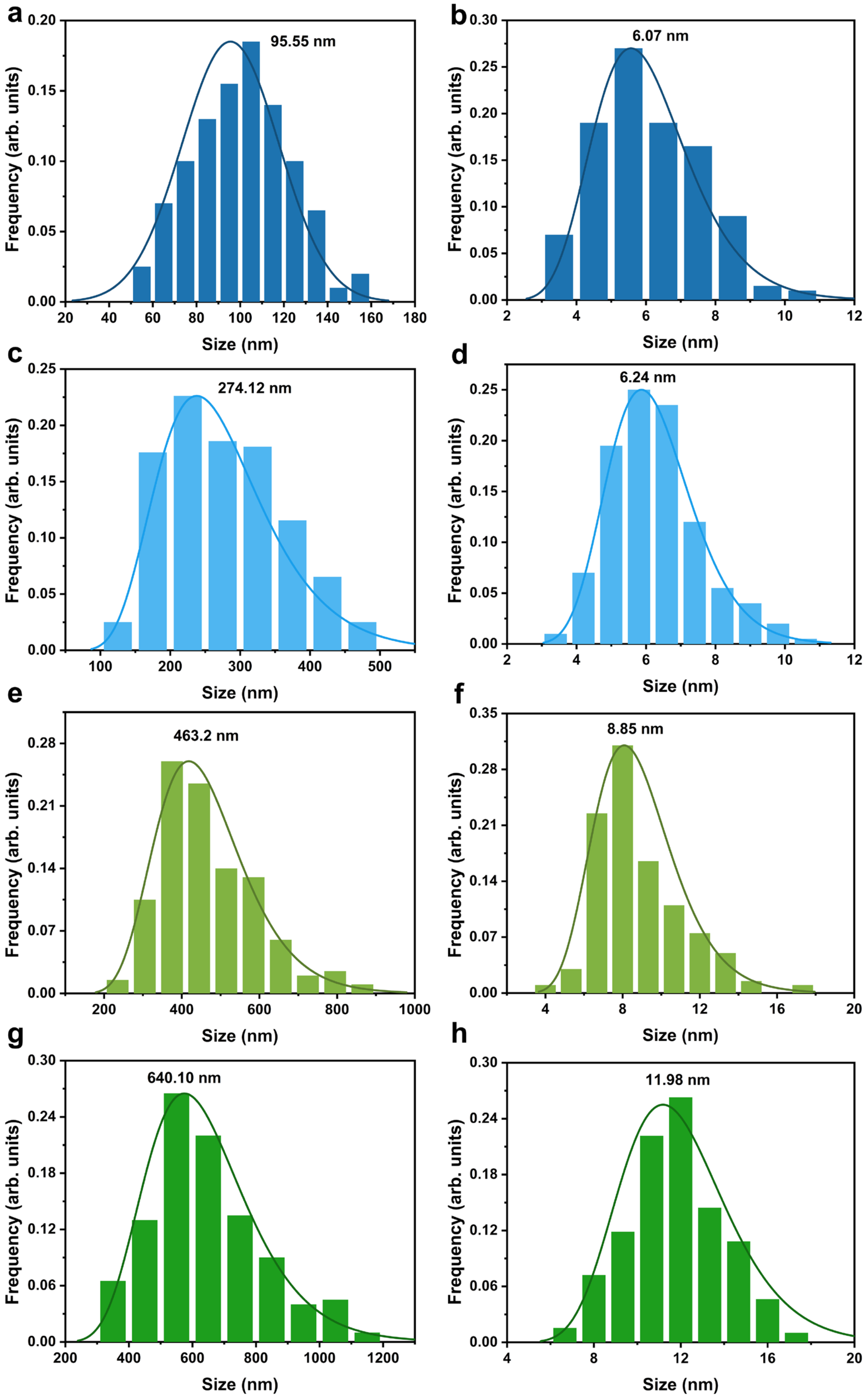

Supplementary Fig. 24 (a, c, e, g) Length and (b, d, f, h) diameter distributions of (a, b) T-IrO_x-400, (c, d) T-IrO_x-500, (e, f) T-IrO_x-600, and (g, h) T-IrO_x-700, respectively. Source data are provided as a Source Data file.

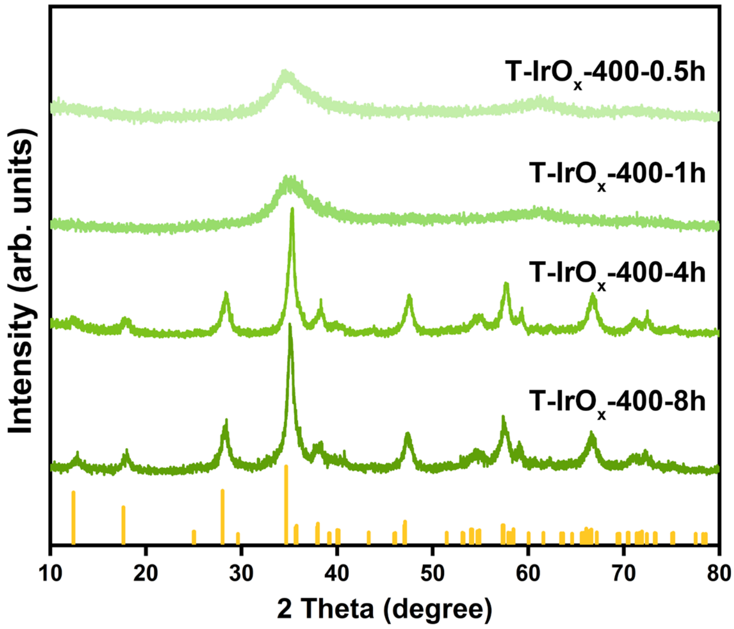

Supplementary Fig. 25 XRD patterns of T-IrO$_x$ synthesized at 400℃ under varying times. Source data are provided as a Source Data file.

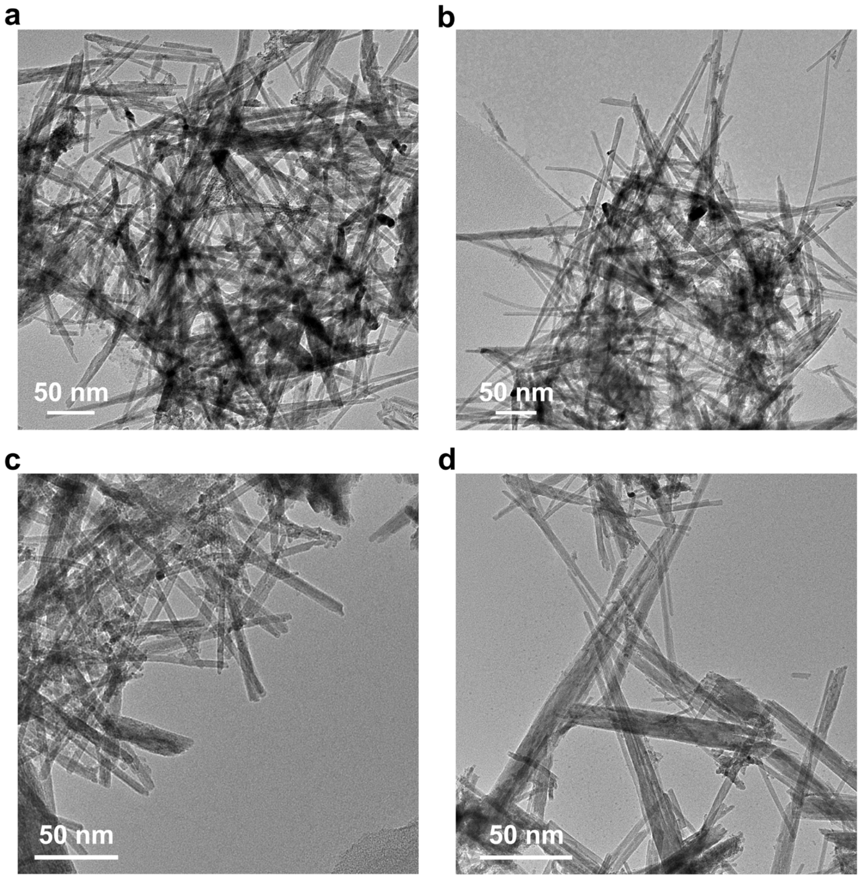

Supplementary Fig. 26 TEM images of (a, b) T-IrO$_x$-400-4h and (c, d) T-IrO$_x$-400-8h, respectively.

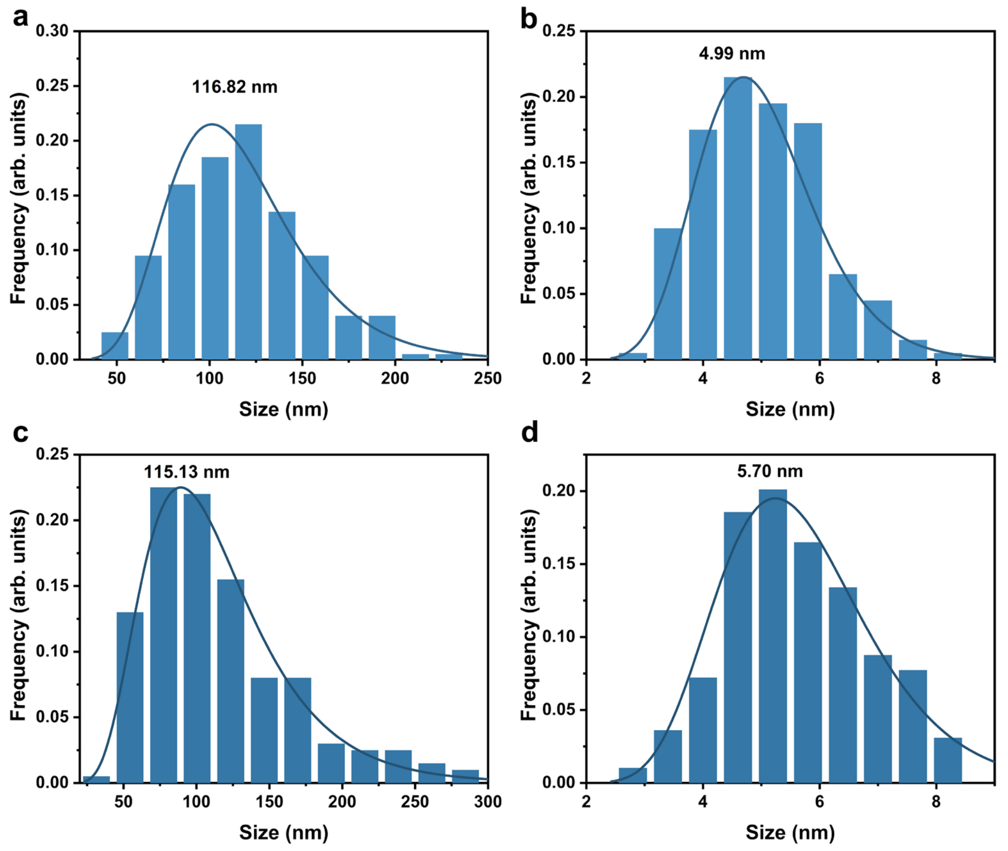

Supplementary Fig. 27 Length and diameter distributions of (a,b) T-IrO_x-400-4h and (c,d) T-IrO_x-400-8h, respectively. Source data are provided as a Source Data file.

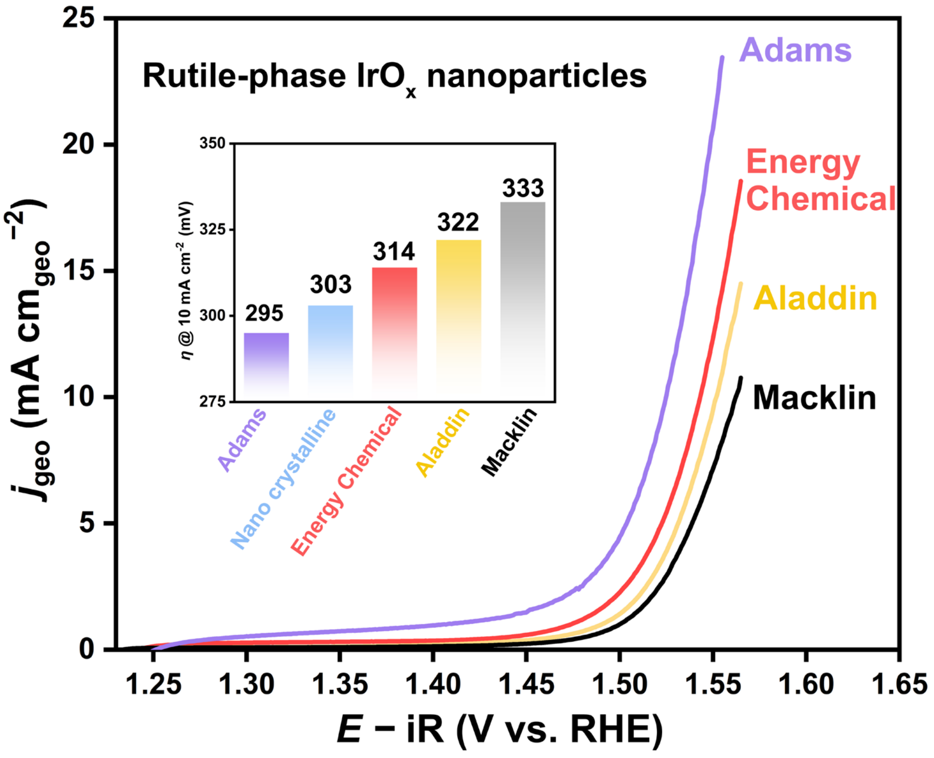

Supplementary Fig. 28 iR-Corrected (85%) polarization curves for OER over R-IrO_x and several commercial rutile IrO_x nanoparticles. The data for commercial nano crystalline IrO_2 samples were adopted from previously reported work[1]. The measurements were performed in O_2-saturated 0.1 M $HClO_4$ (pH 1) at 25 °C, with a scan rate of 1 mV s^{-1}, the catalyst loading of 0.281 mg cm^{-2}, and the O_2 flow rate of 10 mL min^{-1}.The measurements were performed three times. The compensated resistances, measured via the iR compensation function of the electrochemical workstation, were 33.7±0.7 Ω for Energy Chemical, 38.1±0.4 Ω for Aladdin, 37.3±1.0 Ω for Macklin, and 36.2±1.2 Ω for Adams, respectively. Source data are provided as a Source Data file.

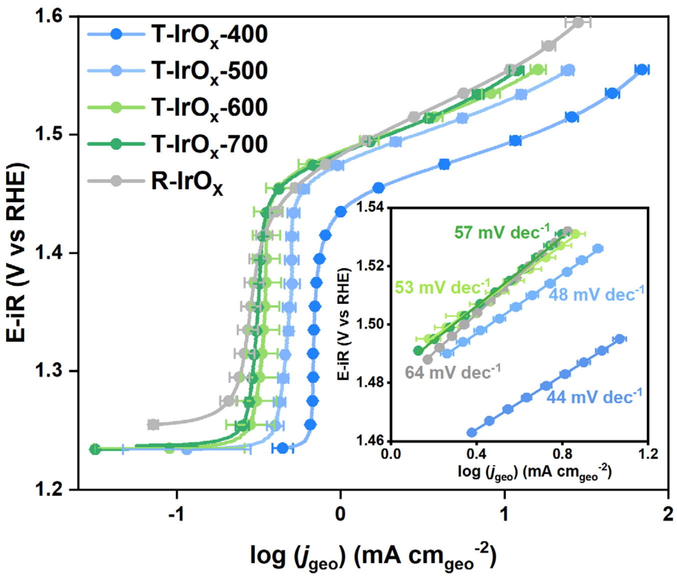

Supplementary Fig. 29 iR-Corrected (85%) tafel slopes of T-IrO_x synthesized at different temperatures and R-IrO_x. Measurements were performed in O_2-saturated 0.1 M $HClO_4$ (pH 1, 25 °C), with a scan rate of 1 mV s^{-1}, the catalyst loading of 0.281 mg cm^{-2}, and the O_2 flow rate of 10 mL min^{-1}.The error bars represent the standard deviation from three measurements. Source data are provided as a Source Data file.

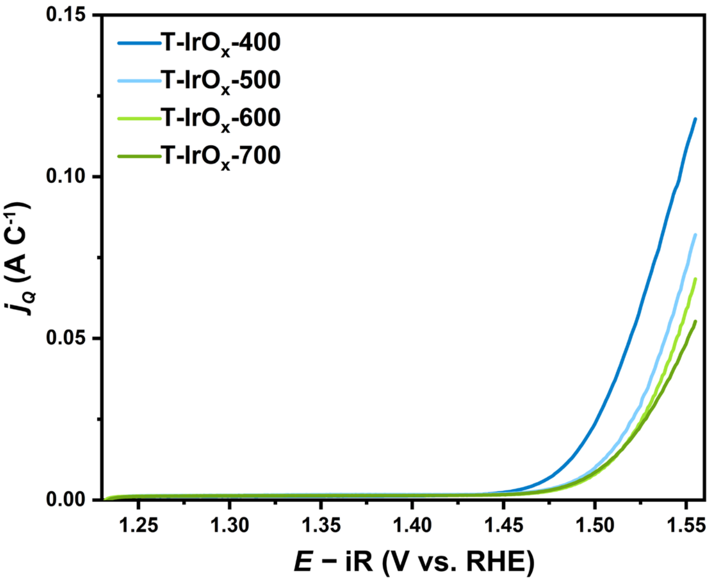

Supplementary Fig. 30 Pseudocapacitive charge-normalized OER activities of T-IrO_x synthesized at different temperatures. The measurements were performed in O_2-saturated 0.1 M $HClO_4$ (pH 1) at 25 °C, with a scan rate of 1 mV s^{-1}, the catalyst loading of 0.281 mg cm^{-2}, and the O_2 flow rate of 10 mL min^{-1}. E−iR presents 85% iR-corrected potentials. The measurements were performed three times. Source data are provided as a Source Data file.

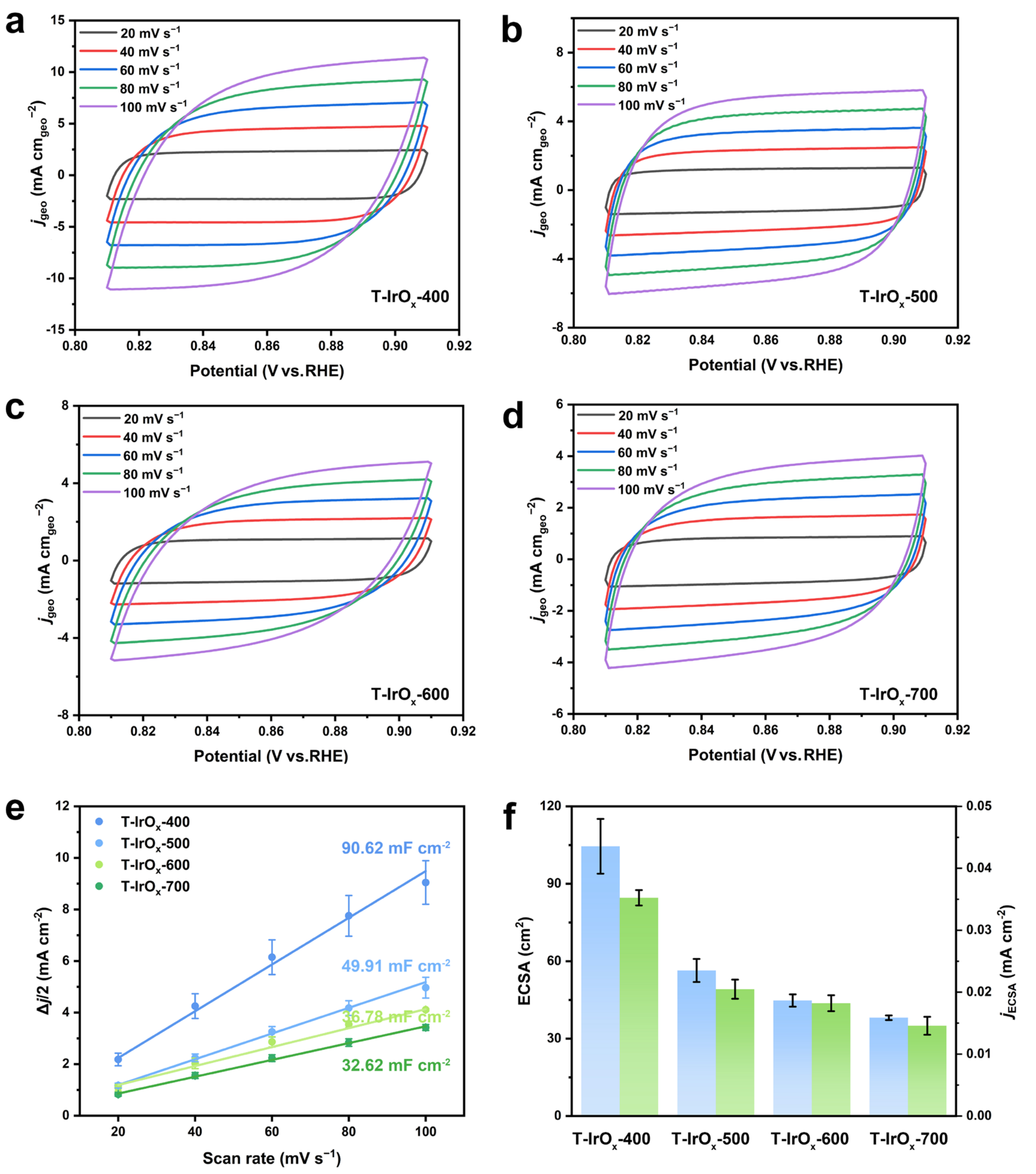

Supplementary Fig. 31 (a-d) Cyclic voltammetry (CV) curves without iR corrected measured in the non-Faradaic region at different scan rates for T-IrO_x synthesized at different temperatures. The measurements were performed in O_2-saturated 0.1 M $HClO_4$ (pH 1) at 25 °C, with the catalyst loading of 0.281 mg cm^{-2}. (e) Double-layer capacitance (C_{dl}) deduced by the fitting slopes of current between anodic and cathodic sweeps versus different scan rates. (f) ECSAs and ECSA-normalized specific activities at 1.54 V RHE over a series of T-IrO_x synthesized at different temperatures. The error bars represent the standard deviation from three measurements. Source data are provided as a Source Data file.

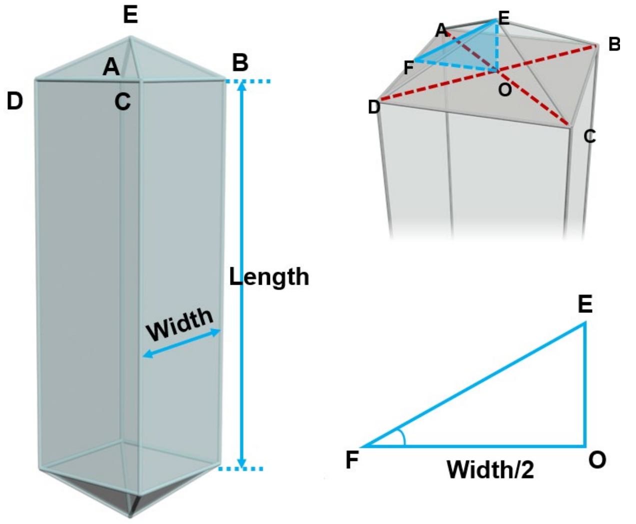

Supplementary Fig. 32 Schematic diagram of the calculation process of mouth area ratio.

The total surface area of each nanorod is composed of the lateral wall area and the mouth area at both ends. We approximate each nanorod as a rectangular cuboid with square cross-section and bipyramidal terminations at both ends, as supported by the symmetry and morphology observed in TEM images. The dominant contribution arises from the four lateral sidewalls, calculated as $A_{side} = 4 \times \text{length} \times \text{width}$. Each nanorod mouth consists of four triangular facets positioned at the tunnel ends. Based on TEM, half of the nanorod length corresponds to one leg (OF) of a right triangle (ΔEFO), with the angle ∠EFO measured to be approximately 30° by statistical analysis of multiple TEM images. Using trigonometric relations, the length of EF can be determined as EF $= 0.5 \times \text{width} / \cos(30°)$. The area of one triangular mouth facet is therefore $A_{mouth,single} = 0.5 \times \text{width} \times EF$. As there are four equivalent facets at each end and two ends per nanorod, the total mouth area is $A_{mouth} = 2 \times \text{width}^2 / \cos(30°)$. The mouth area ratio is then defined as Mouth area ratio $= A_{mouth} / (A_{mouth} + A_{side})$.

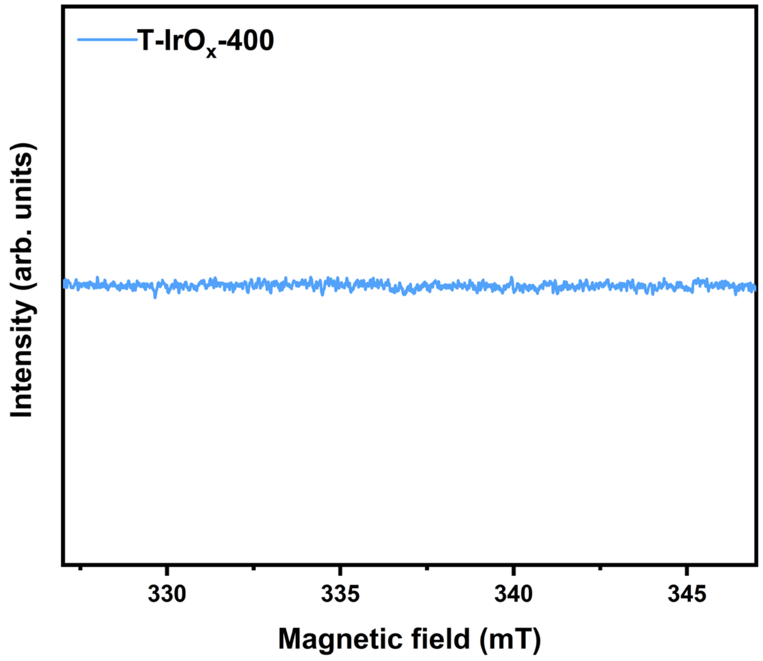

Supplementary Fig. 33 EPR spectrum of T-IrO$_x$-400. Source data are provided as a Source Data file.

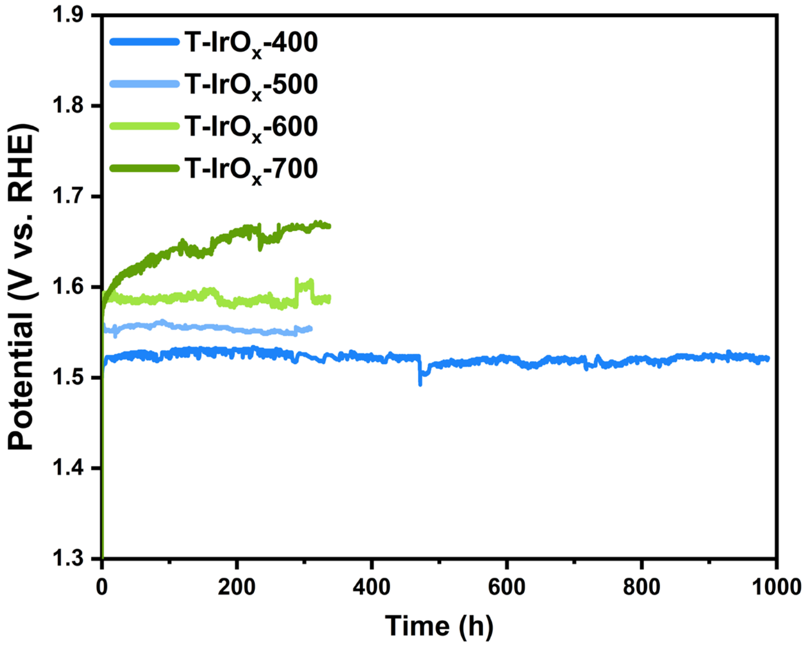

Supplementary Fig. 34 Chronopotentiometric curves of T-IrO$_x$-400, T-IrO$_x$-500, T-IrO$_x$-600, T-IrO$_x$-700 at j_{geo} = 10 mA cm_{geo}^{-2} without iR compensation. The measurements were performed in O_2-saturated 0.1 M $HClO_4$ (pH 1) at 25 °C, with the catalyst loading of 0.667 mg cm^{-2}, and the O_2 flow rate of 10 mL min^{-1}. The measurements were performed once. Source data are provided as a Source Data file.

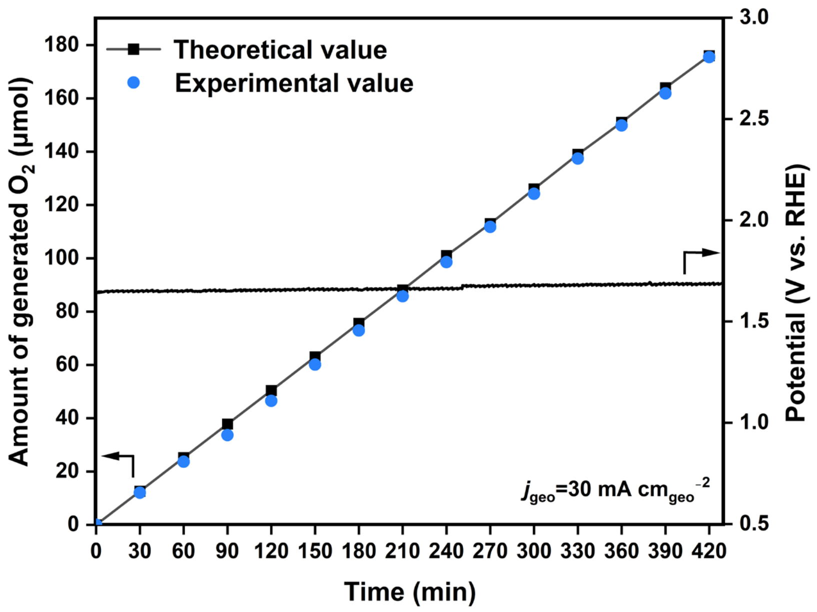

Supplementary Fig. 35 Faradaic efficiency of oxygen generation without iR compensation over T-IrO_x-400 at a current density of 30 mA cm_{geo}^{-2}. The measurements were performed in O_2-saturated 0.1 M $HClO_4$ (pH 1) at 25 °C, with the catalyst loading of 0.444 mg cm^{-2}.The measurements were performed once. Source data are provided as a Source Data file.

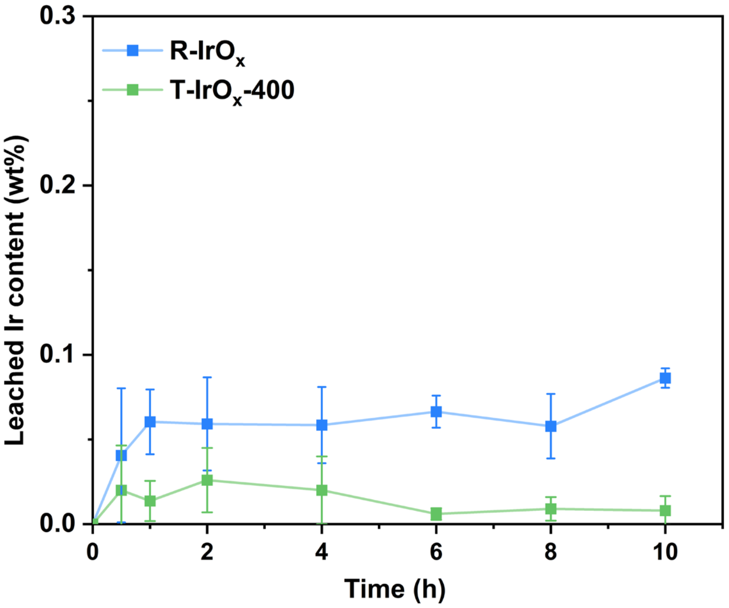

Supplementary Fig. 36 Leached Ir content in the electrolyte from T-IrO_x-400 and R-IrO_x during 10 hours of catalysis. The measurements were performed in O_2-saturated 0.1 M $HClO_4$ (pH 1) at 25 °C, with the catalyst loading of 10 mg cm^{-2}, at an applied potential of 1.586 V vs RHE. The error bars represent the standard deviation from three measurements. Source data are provided as a Source Data file.

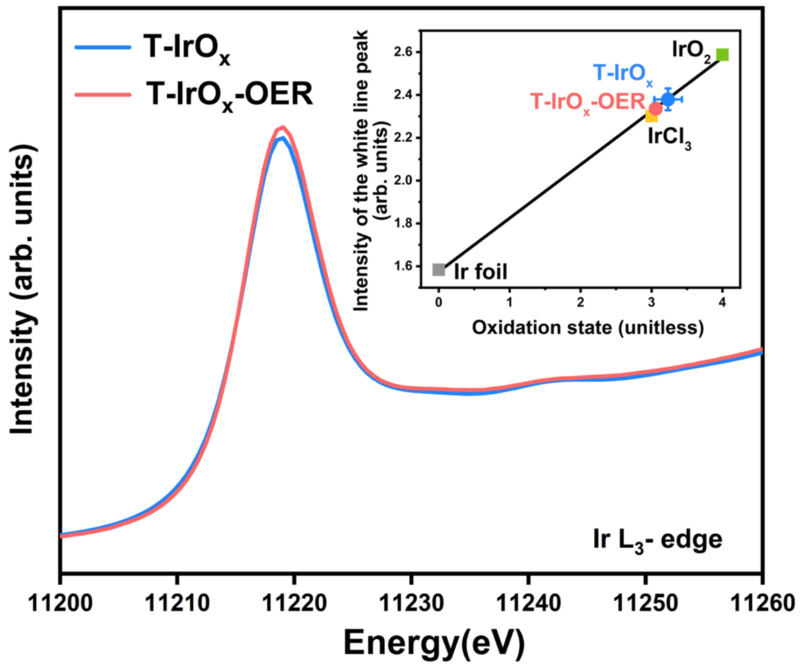

Supplementary Fig. 37 Normalized XANES spectra of T-IrO_x and T-IrO_x-OER. The inset shows the fitted iridium oxidation states derived from the L_3-edge white line intensity for T-IrO_x, T-IrO_x-OER, IrO_2, $IrCl_3$, and Ir foil. Source data are provided as a Source Data file.

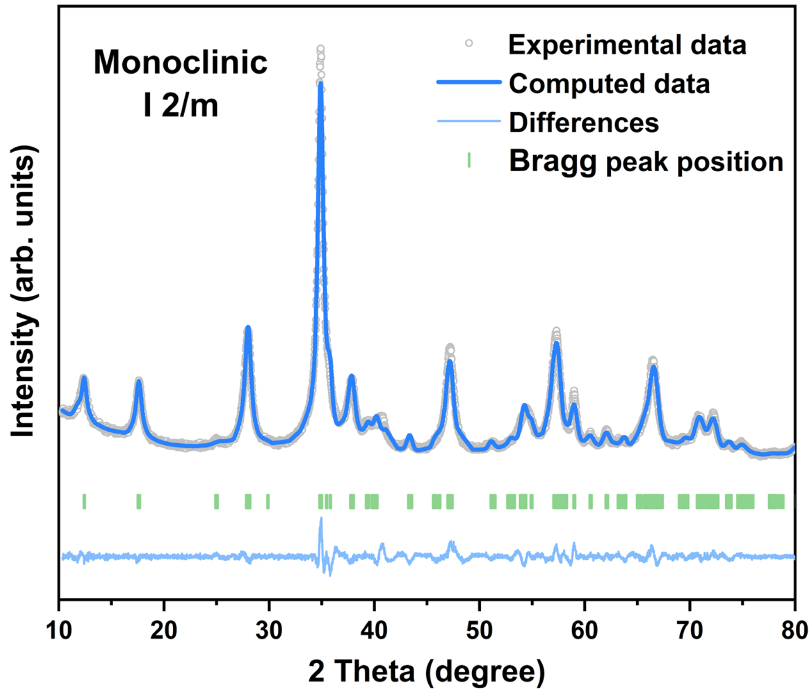

Supplementary Fig. 38 Rietveld refinement of XRD pattern for T-IrO_x-OER. Source data are provided as a Source Data file.

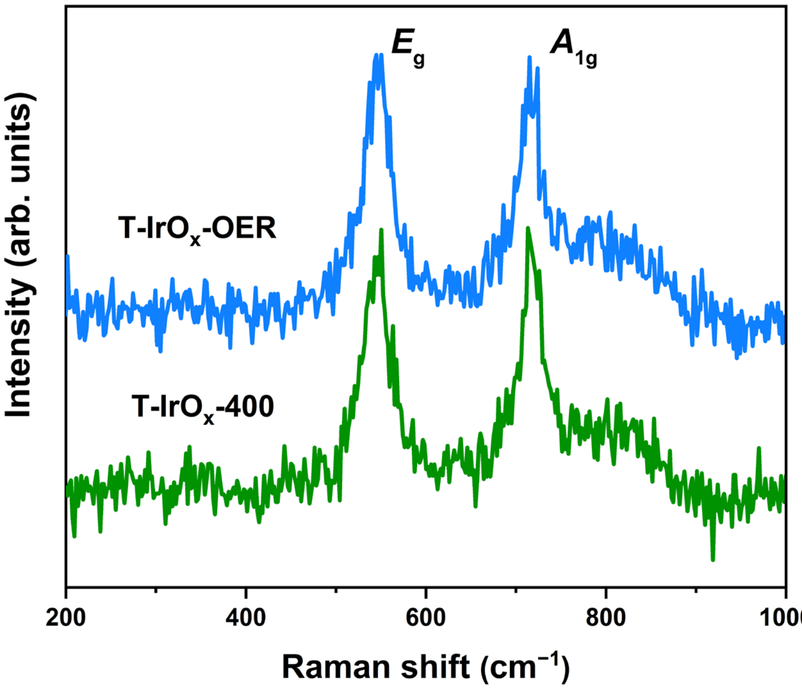

Supplementary Fig. 39 Raman spectra of T-IrO_x-400 and T-IrO_x-OER. The peaks at approximately 550 cm^{-1} and 720 cm^{-1} correspond to the bending vibration (E_g mode) and symmetric stretching vibration (A_{1g} mode) of the Ir-O bonds, respectively. Source data are provided as a Source Data file.

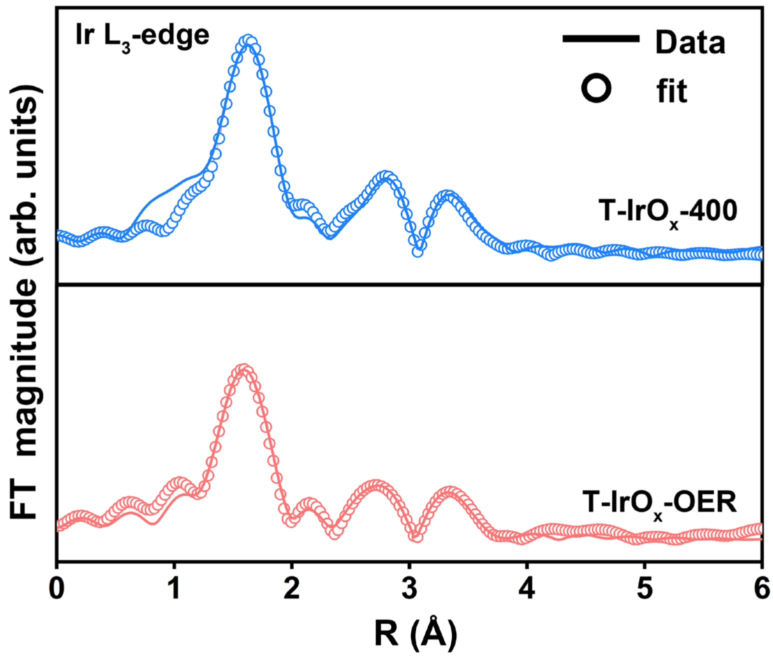

Supplementary Fig. 40 Fourier-transformed Ir L_3-edge EXAFS spectra of T-IrO_x and T-IrO_x-OER. Source data are provided as a Source Data file.

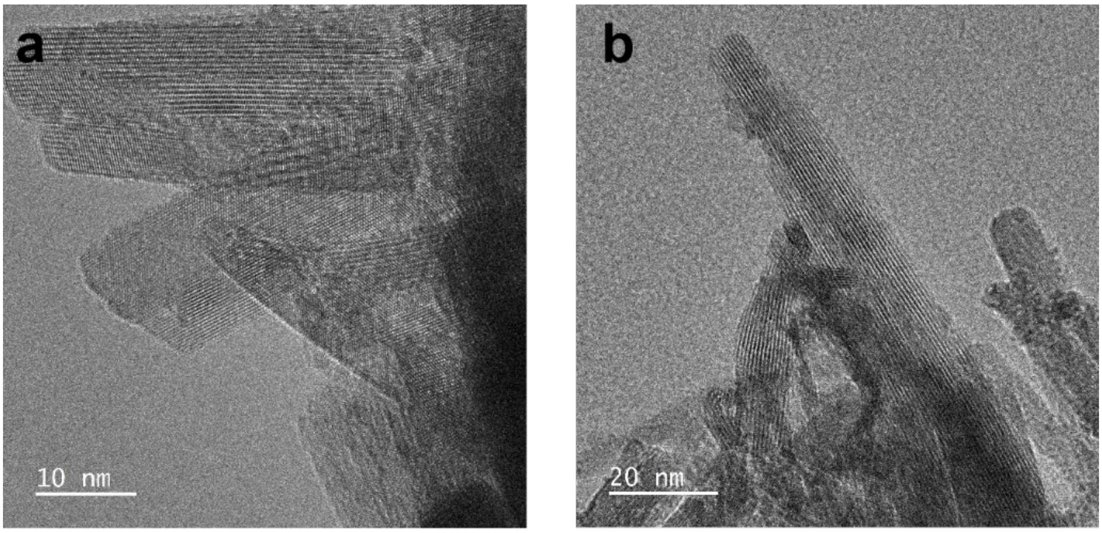

Supplementary Fig. 41 The HRTEM images of T-IrO_x-400-OER.

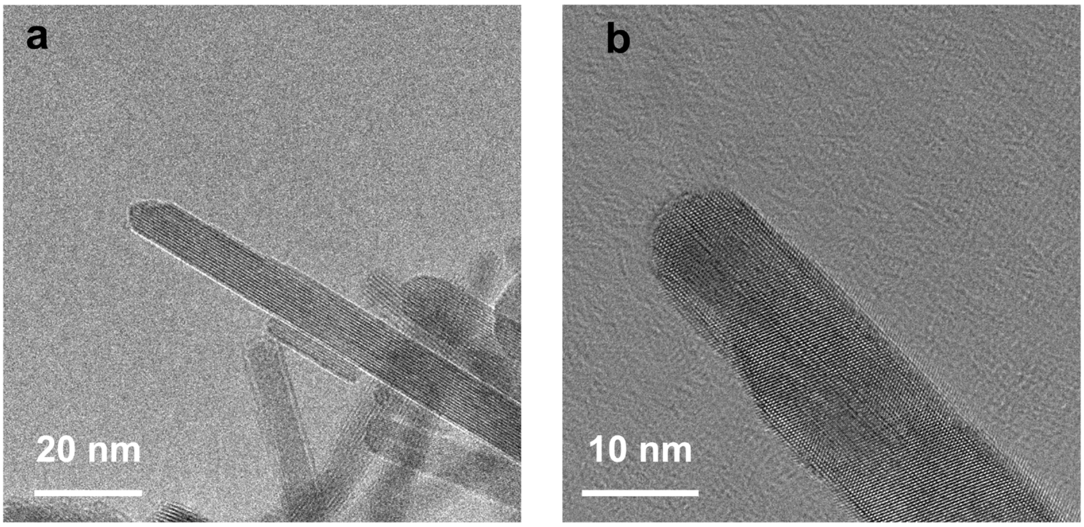

Supplementary Fig. 42 The HRTEM images of T-IrO_x-500-OER.

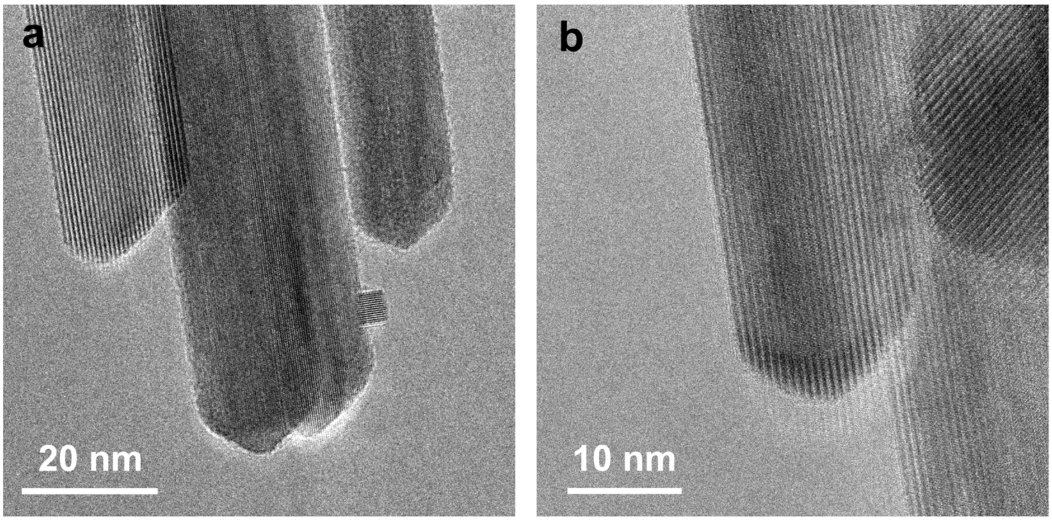

Supplementary Fig. 43 The HRTEM images of T-IrO_x-600-OER.

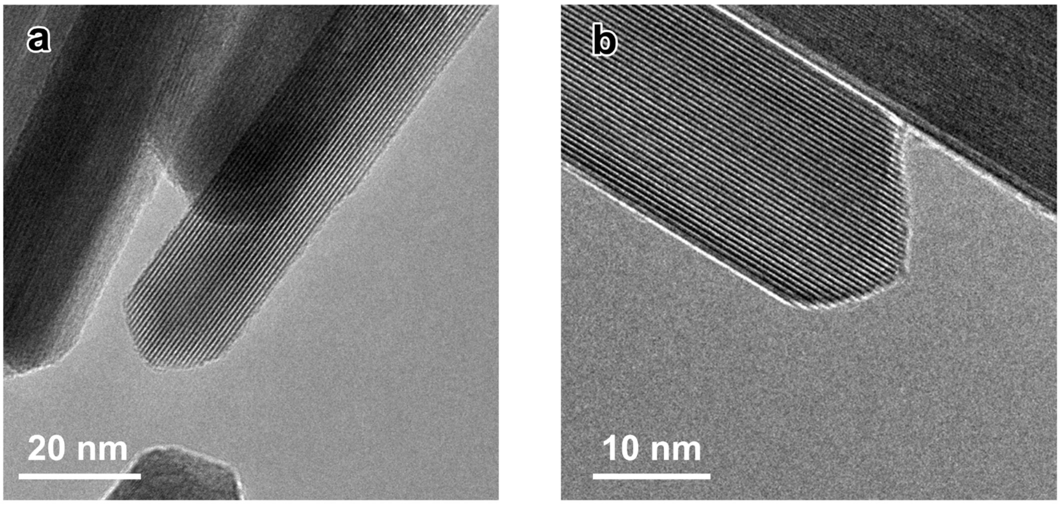

Supplementary Fig. 44 The HRTEM images of T-IrO_x-700-OER.

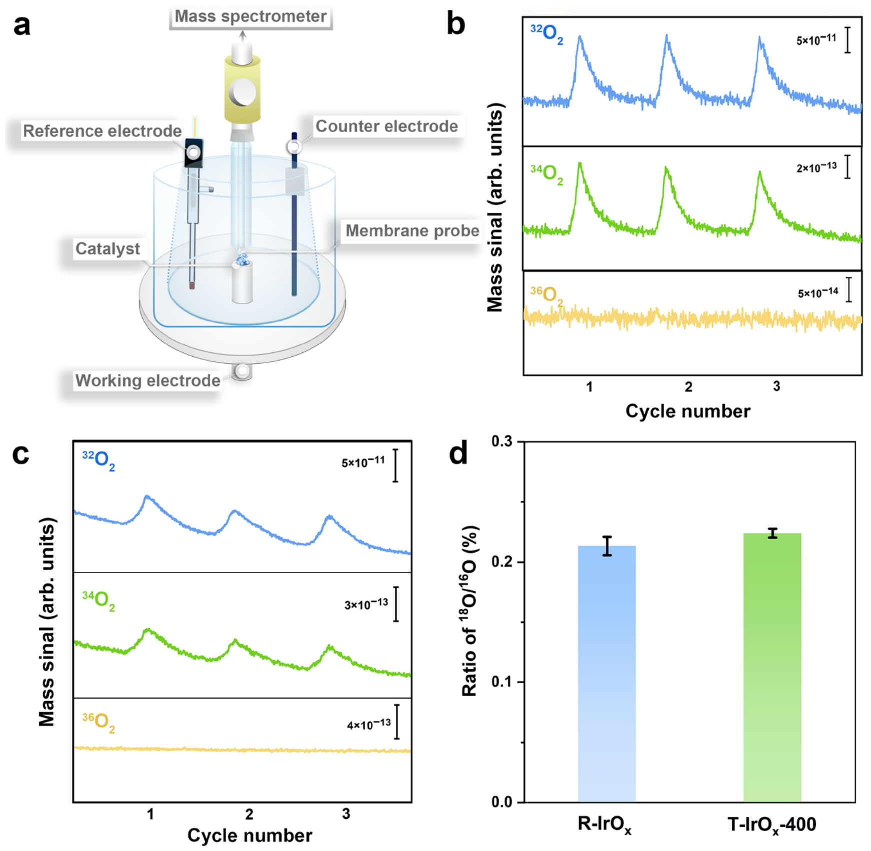

Supplementary Fig. 45 (a) Schematic illustration of the in situ electrochemical differential mass spectrometry (DEMS) testing setup. DEMS signals of O_2 products for the OER process of ^{18}O-labeled (b) T-IrO$_x$-400 and (c) R-IrO$_x$. (d) Ratio of $^{18}O/^{16}O$ for T-IrO$_x$-400 and R-IrO$_x$. The measurements were performed in 0.1 M $HClO_4$ (pH 1) at 25 °C, with the catalyst loading of 0.212 mg cm^{-2}. The error bars represent the standard deviation from three measurements. Source data are provided as a Source Data file.

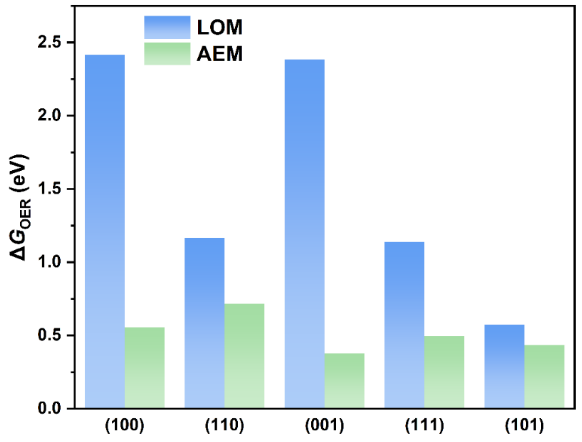

Supplementary Fig. 46 Theoretical OER activities for different crystal planes of T-IrO$_x$ through LOM reaction pathway and AEM reaction pathway. Source data are provided as a Source Data file.

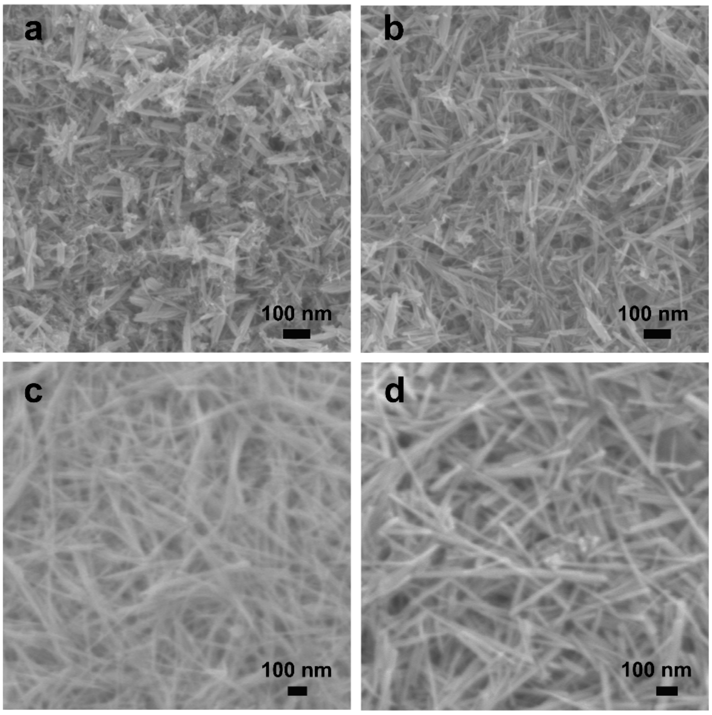

Supplementary Fig. 47 SEM images of (a) T-IrO_x-400, (b) T-IrO_x-500, (c) T-IrO_x-600, and (d) T-IrO_x-700 based CCMs, respectively.

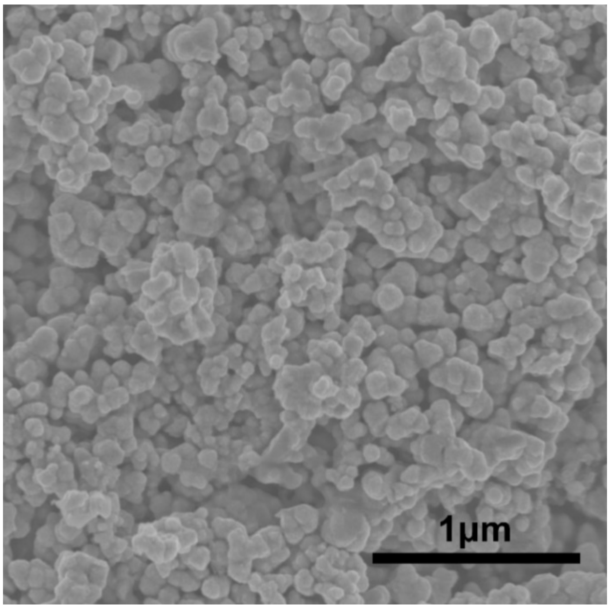

Supplementary Fig. 48 SEM image of the R-IrO_x-based CCM.

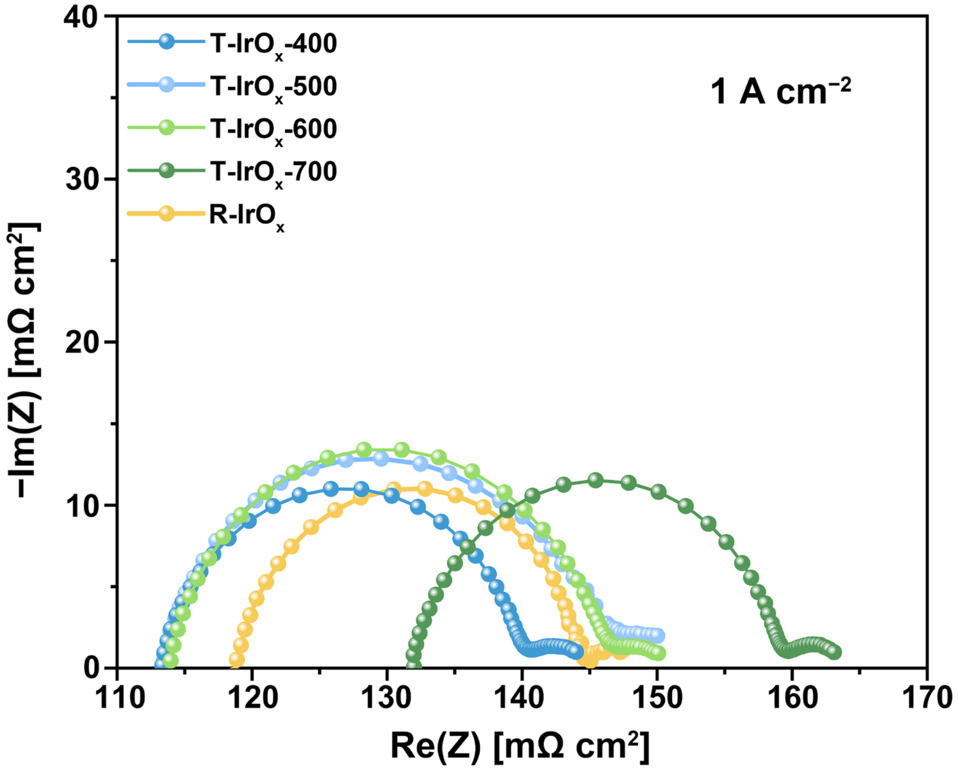

Supplementary Fig. 49 The EIS result of four T-IrO_x- and R-IrO_x-based CCMs. The measurements were performed three times. Source data are provided as a Source Data file.

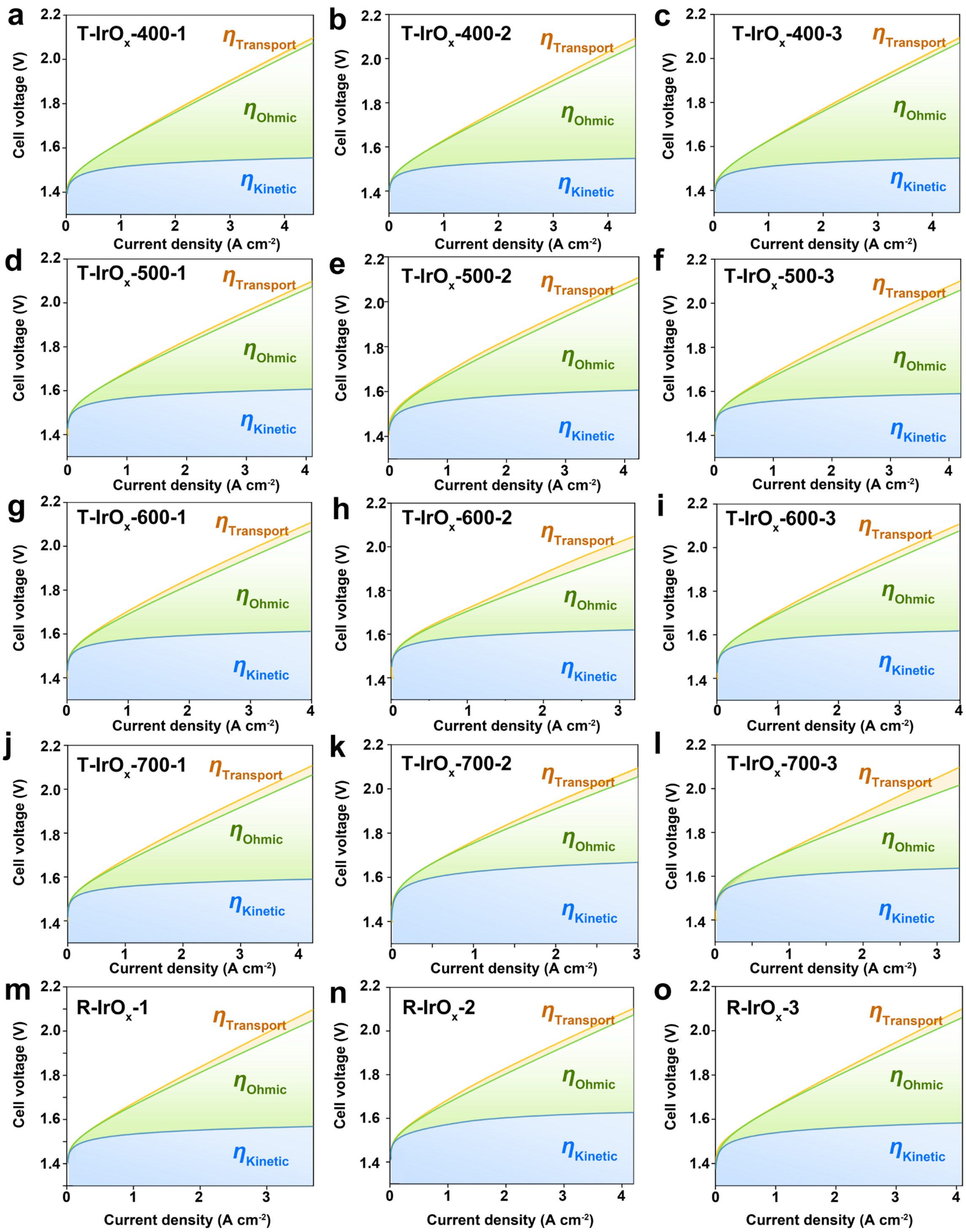

Supplementary Fig. 50 The breakdowns of voltage losses without iR-corrected for PEMWEs based on (a-c) T-IrO_x-400, (d-f) T-IrO_x-500, (g-i) T-IrO_x-600, (j-l) T-IrO_x-700, (m-o) R-IrO_x by three parallel experiments. Source data are provided as a Source Data file.

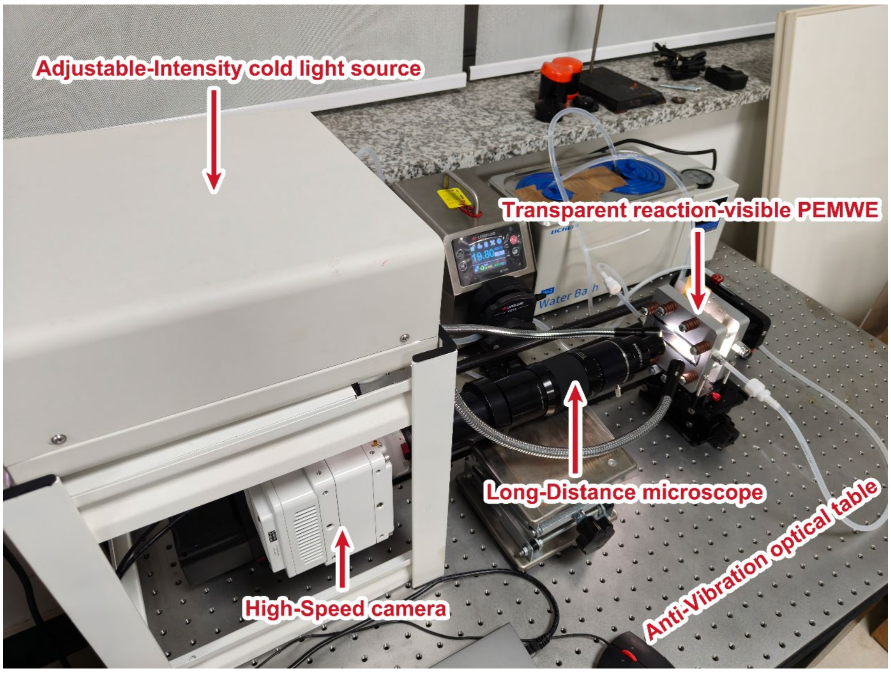

Supplementary Fig. 51 The photo of HMVS setup used for observing mass transport in transparent reaction-visible PEMWE.

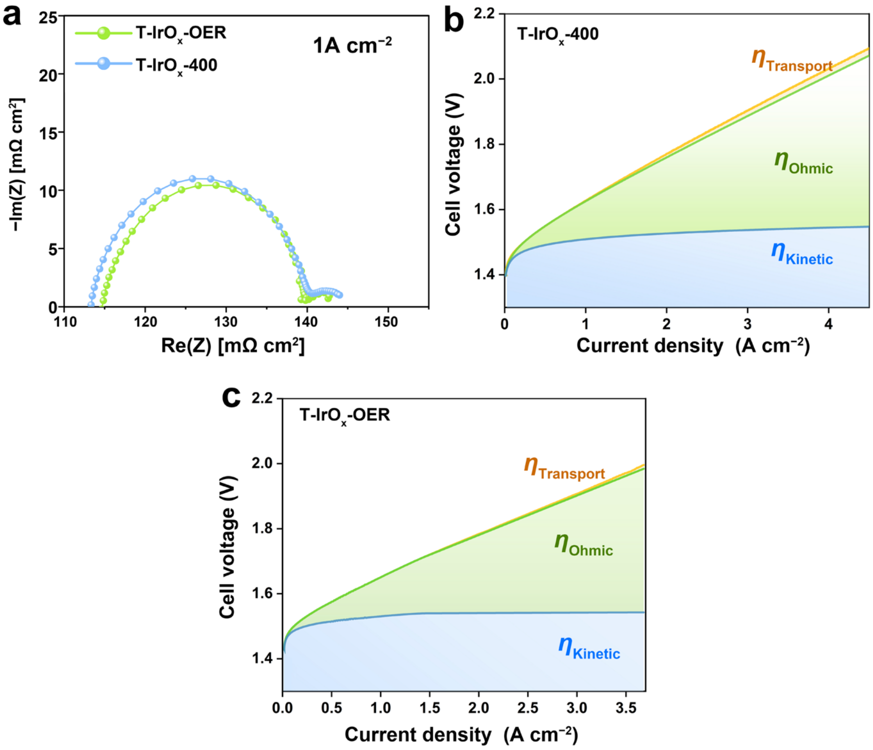

Supplementary Fig. 52 (a) The EIS result of PEMWEs based on T-IrO_x-400 and T-IrO_x-OER-1000h. The breakdowns of voltage losses without iR-corrected of PEMWEs based on (b) T-IrO_x-400 and (c) T-IrO_x-OER-1000h. The measurements were performed once. Source data are provided as a Source Data file.

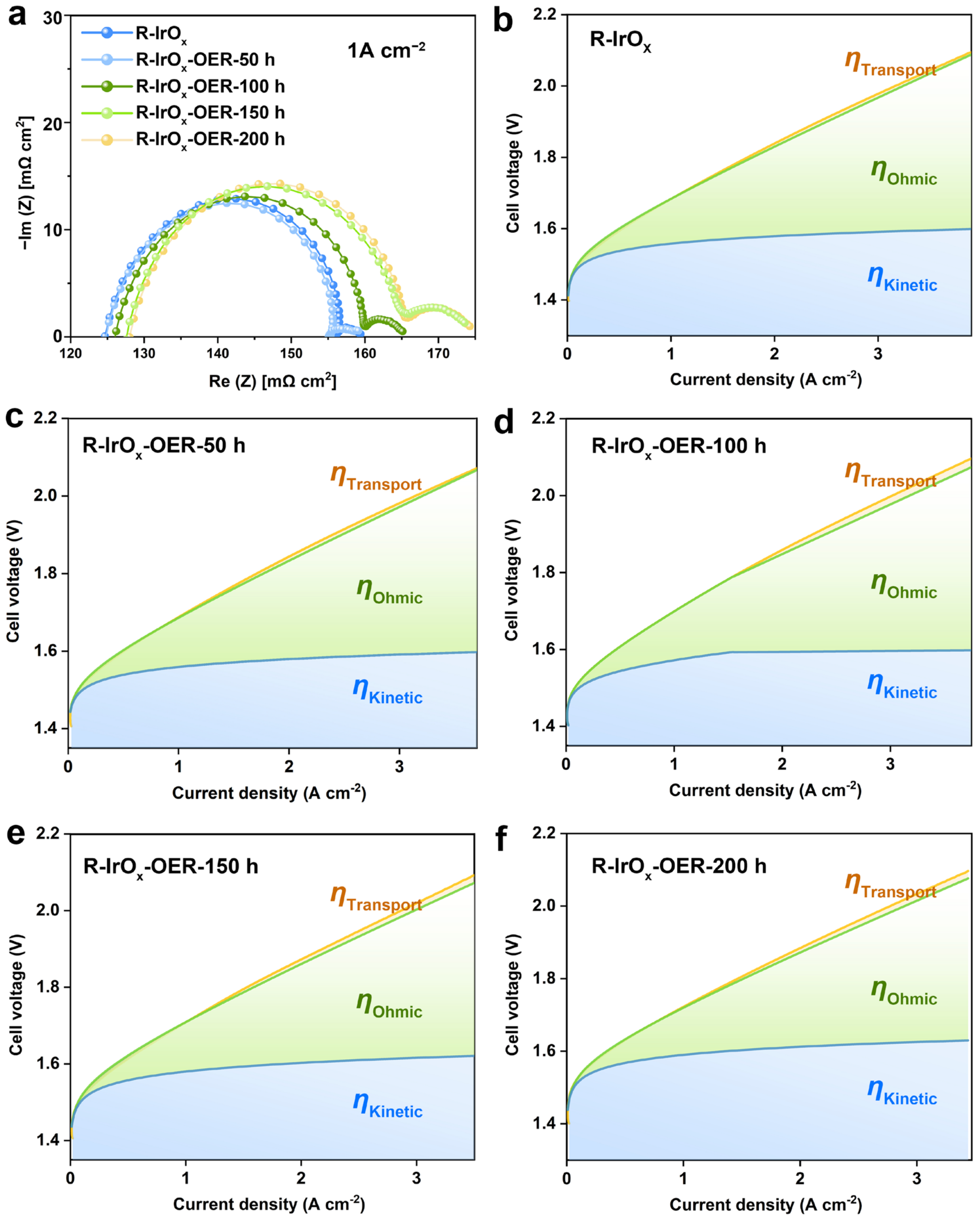

Supplementary Fig. 53 (a) The EIS result of PEMWEs based on R-IrO_x, R-IrO_x-OER-50h, R-IrO_x-OER-100h, R-IrO_x-OER-150h and R-IrO_x-OER-200h. The breakdowns for voltage losses without iR-corrected of PEMWEs based on (b) R-IrO_x, (c) R-IrO_x-OER-50h, (d) R-IrO_x-OER-100h, (e) R-IrO_x-OER-150h, and (f) R-IrO_x-OER-200h. The measurements were performed once. Source data are provided as a Source Data file.

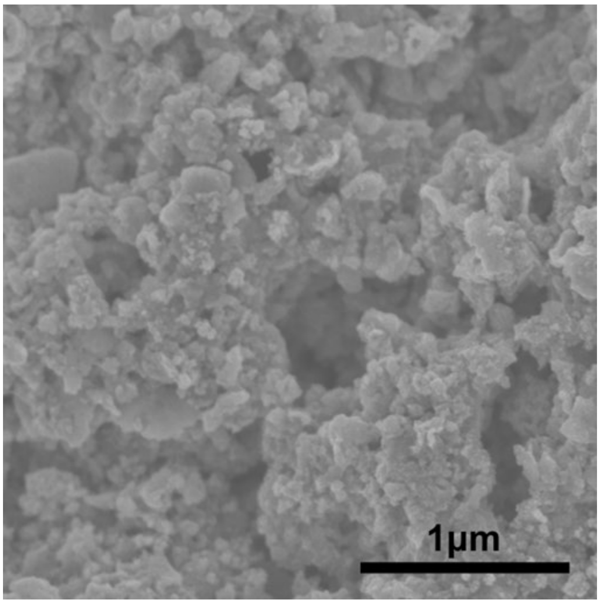

Supplementary Fig. 54 SEM image of the R-IrO_x-based CCM after operation.

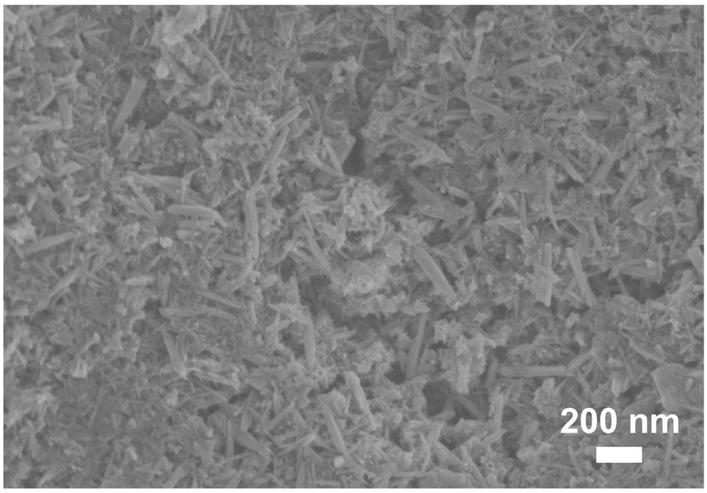

Supplementary Fig. 55 SEM images of the T-IrO_x-based CCM after operation.

Supplementary Table 1. Calculated energy change ($\Delta E_{insertion}$) for water molecule insertion into tunnel structures. Note: For 1×2 and 1×2' tunnels, stable incorporation of water molecules is not feasible.

**Tunnel type**	**$\Delta E_{insertion}$ (eV/atom)**
2×2	-0.032
2×3	-0.024
3×3	-0.032

Supplementary Table 2. Calculated OER overpotential (η_{OER}) for the inner active sites of 2×2, 2×3, and 3×3 tunnel iridium oxides. For cases not explicitly showing an overpotential, the key OER intermediate OOH* could not be stabilized. All computational files are provided in the Supplementary Data 1.

**Tunnel type**	**(*hkl*)**	**η_{OER} (V)**
2×2	(100)	2.450
2×2	(101)	3.238
3×3	(011)	1.573

Supplementary Table 3. The Gibbs free energy for each step of AEM and the OER overpotential (η_{OER}) of five tunnel iridium oxides.

**Tunnel type**	**(*hkl*)**	**ΔG_1 (eV)**	**ΔG_2 (eV)**	**ΔG_3 (eV)**	**ΔG_4 (eV)**	**η_{OER} (V)**
1×2	(001)	0.647	1.285	1.728	1.260	0.498
1×2	(010)	0.211	1.167	1.878	1.665	0.648
1×2	(011)	0.264	1.554	1.627	1.476	0.397
1×2	(100)	-0.035	1.550	1.828	1.577	0.598

1×2	(101)	0.755	1.450	1.549	1.166	0.319
1×2	(110)	0.220	1.159	1.912	1.629	0.682
1×2	(111)	0.310	1.286	1.786	1.537	0.556
1×2'	(001)	0.829	1.312	1.637	1.142	0.407
1×2'	(010)	-0.127	1.487	1.581	1.979	0.749
1×2'	(011)	0.226	1.312	1.718	1.664	0.488
1×2'	(100)	0.697	1.220	1.755	1.248	0.525
1×2'	(101)	0.361	1.249	1.762	1.548	0.532
1×2'	(110)	-0.025	1.299	1.600	2.046	0.816
1×2'	(111)	0.452	1.559	1.602	1.307	0.372
2×2	(001)	0.748	1.303	1.607	1.262	0.377
2×2	(100)	0.053	1.510	1.571	1.786	0.556
2×2	(101)	0.734	1.360	1.664	1.162	0.434
2×2	(110)	0.461	1.005	1.946	1.508	0.716
2×2	(111)	0.799	1.336	1.727	1.059	0.497
2×3	(001)	0.732	1.326	1.732	1.130	0.502
2×3	(010)	0.397	1.045	1.937	1.541	0.707
2×3	(011)	0.486	1.547	1.516	1.371	0.317
2×3	(100)	0.078	1.490	1.902	1.450	0.672
2×3	(101)	0.856	1.243	1.758	1.062	0.528
2×3	(110)	0.220	1.163	1.963	1.574	0.733
2×3	(111)	0.626	1.268	1.687	1.339	0.457
3×3	(001)	0.531	1.271	1.666	1.452	0.436

3×3	(010)	0.302	1.176	1.933	1.509	0.703
3×3	(011)	0.642	1.438	1.615	1.225	0.385
3×3	(110)	0.112	1.478	1.337	1.992	0.762
3×3	(111)	0.571	1.356	1.612	1.382	0.382

Supplementary Table 4. The Ir 5d-band center, O 2p-band center, charge transfer energy (ε_{d-p}), −ICOHP of Ir-O and Bader charge of oxygen atoms in five tunnel iridium oxides.

**Tunnel type**	**(*hkl*)**	**Ir d-band center (eV)**	**O p-band center (eV)**	**ε_{d-p} (eV)**	**−ICOHP**	**O Bader charge (e atom^{-1})**
1×2	(001)	-2.852	-3.739	0.887	3.466	-0.753
1×2	(010)	-2.604	-3.857	1.253	3.323	-0.777
1×2	(011)	-2.884	-3.531	0.647	3.504	-0.720
1×2	(100)	-2.605	-4.088	1.483	3.131	-0.809
1×2	(101)	-2.846	-3.895	1.049	3.419	-0.772
1×2	(110)	-2.708	-4.096	1.388	3.275	-0.777
1×2	(111)	-2.911	-3.653	0.742	3.505	-0.735
1×2'	(001)	-2.769	-3.353	0.584	3.351	-0.765
1×2'	(010)	-2.614	-3.760	1.146	3.173	-0.820
1×2'	(011)	-2.897	-3.179	0.282	3.409	-0.732
1×2'	(100)	-2.425	-3.232	0.807	3.298	-0.807
1×2'	(101)	-2.763	-3.423	0.660	3.239	-0.766

1×2'	(110)	-2.621	-3.843	1.222	3.175	-0.806
1×2'	(111)	-2.768	-3.229	0.461	3.422	-0.755
2×2	(001)	-2.808	-3.737	0.929	3.407	-0.760
2×2	(100)	-2.373	-3.674	1.301	3.243	-0.780
2×2	(101)	-2.841	-3.754	0.913	3.362	-0.748
2×2	(110)	-2.490	-3.795	1.305	3.249	-0.777
2×2	(111)	-2.802	-3.613	0.811	3.413	-0.730
2×3	(001)	-2.830	-3.865	1.035	3.360	-0.760
2×3	(010)	-2.698	-4.172	1.474	3.274	-0.810
2×3	(011)	-2.787	-3.743	0.956	3.363	-0.750
2×3	(100)	-2.421	-4.075	1.654	3.177	-0.808
2×3	(101)	-2.841	-3.773	0.932	3.393	-0.751
2×3	(110)	-2.579	-4.036	1.457	3.194	-0.792
2×3	(111)	-2.790	-3.795	1.005	3.351	-0.761
3×3	(001)	-2.729	-3.956	1.227	3.217	-0.767
3×3	(010)	-2.426	-4.447	2.021	2.923	-0.843
3×3	(011)	-2.703	-3.711	1.008	3.318	-0.757
3×3	(110)	-2.323	-4.315	1.992	2.925	-0.831
3×3	(111)	-2.653	-4.013	1.360	3.211	-2.653

Supplementary Table 5. Rietveld refinement of XRD results for T-IrO_x-400.

	**Space group**	**Lattice constant**	**Rp (%)**	**Rwp (%)**	χ^2
T-IrO_x-400	I 2/m	*a* = 10.14	5.71	7.61	2.39

		b = 3.14<br>c = 10.08<br>α = γ =90°<br>β = 89.85°			
T-IrO_x-OER	I 2/m	a = 10.03<br>b = 3.13<br>c = 10.13<br>α = γ = 90°<br>β = 90.33°	4.55	5.97	4.02

Supplementary Table 6. EXAFS fitting parameters at the Ir L_3-edge for T-IrO_x synthesized at different temperatures.

Sample	Shell	N^a	R(Å)^b	σ^2(Å^2)^c	ΔE_0 (eV)^d	R factor
T-IrO_x-400	Ir-O	6.3±0.3	2.00±0.01	0.0038	14.0±0.7	0.0105
	Ir-Ir	6.4±1.0	3.13±0.01	0.0066		
	Ir-Ir1	7.8±2.1	3.54±0.02	0.0075		
	Ir-O1	8.5±2.0	3.63±0.02	0.0017		
T-IrO_x-500	Ir-O	6.5±0.3	2.00±0.01	0.0030	13.3±0.8	0.0111
	Ir-Ir	8.1±1.2	3.14±0.01	0.0071		
	Ir-Ir1	11.2±2.3	3.54±0.02	0.0068		
	Ir-O1	8.9±2.0	3.62±0.02	0.0001		
T-IrO_x-600	Ir-O	6.4±0.3	2.00±0.01	0.0031	14.2±0.7	0.0096
	Ir-Ir	7.3±1.0	3.14±0.01	0.0063		
	Ir-Ir1	10.8±1.9	3.53±0.01	0.0064		
	Ir-O1	8.9±1.8	3.62±0.02	0.0003		
T-IrO_x-700	Ir-O	6.3±0.3	2.00±0.01	0.0034	13.7±0.8	0.0113
	Ir-Ir	7.3±1.1	3.13±0.01	0.0067		
	Ir-Ir1	10.4±1.9	3.53±0.02	0.0064		
	Ir-O1	8.4±1.9	3.62±0.02	0.0006		
T-IrO_x-OER	Ir-O	5.9±0.2	1.99±0.01	0.0018	10.6±0.8	0.0101
	Ir-Ir	5.3±0.9	3.12±0.01	0.0050		
	Ir-Ir1	7.9±2.2	3.55±0.02	0.0067		
	Ir-O1	9.1±2.1	3.63±0.03	0.0012		

^{a}*CN*: coordination numbers; ^{b}*R*: bond distance; $^c\sigma^2$: Debye-Waller factors; ^{d} ΔE_0: the inner potential correction. *R* factor: goodness of fit. S_0^2 was set to 0.75, according to the experimental EXAFS fit of Ir foil reference by fixing CN as the known crystallographic value.

Supplementary Table 7. The surface energy of 2×2 tunnel.

(*hkl*)	**Surface energy (J m^{-2})**
(001)	1.499
(100)	1.066
(101)	1.393
(110)	1.204
(111)	1.546

Supplementary Table 8. Comparison of OER activities and stabilities of with representative Ir-based electrocatalysts.

**Catalyst**	**Electrolyte**	***η* at 10 mA cm^{-2} (mV)**	**Stability**
T-IrO_x-400	0.1 M $HClO_4$	263	1.5 V@10 mA cm^{-2}@1000 h
T-IrO_x-500	0.1 M $HClO_4$	282	-
T-IrO_x-600	0.1 M $HClO_4$	298	-
T-IrO_x-700	0.1 M $HClO_4$	312	-
nano-crystalline R-IrO_2	0.5 M H_2SO_4	303	-
3C-$SrIrO_3$[2]	0.5 M H_2SO_4	270	1.52 V@ 10 mA cm^2@30 h
6H-$SrIrO_3$[2]	0.5 M H_2SO_4	248	1.48 V@ 10 mA cm^{-2}@30 h
$Y_2Ir_2O_7$[3]	0.1 M $HClO_4$	350	-
Ca_2IrO_4[4]	0.1 M $HClO_4$	-	-
$W_{1-x}Ir_xO_{3-\delta}$[5]	1 M H_2SO_4	370	1.7 V@ 10 mA cm^{-2}@0.56 h
Ba_2YIrO_6[6]	0.1 M $HClO_4$	370	1.59 V@ 10 mA cm^{-2}@1 h
Li@IrO_x[7]	0.5 M H_2SO_4	270	1.55 V@ 10 mA cm^{-2}@10 h

La_3IrO_7[8]	0.1 M $HClO_4$	296	1.6 V@ 10 mA cm^{-2}@16.7 h
$Pr_2Ir_2O_7$[9]	0.1 M $HClO_4$	290	1.58 V@ 10 mA cm^{-2}@2.8 h
e-H-Li-213[10]	0.5 M H_2SO_4	277	-
Sr_4IrO_6[11]	0.1 M $HClO_4$	287	1.58 V@ 10 mA/cm^{-2}@6 h

Supplementary Table 9. The OER overpotential (η_{OER}') of 2×2 tunnel for LOM.

**(*hkl*)**	**$\eta_{OER'}$ (V)**
(001)	2.383
(100)	2.415
(101)	0.573
(110)	1.167
(111)	1.138

Supplementary Table 10. Ir and Pt mass loadings of T-IrO_x-based CCMs.

**Test number**	**Ir (mg cm^{-2})**	**Pt (mg cm^{-2})**
1	0.29	0.22
2	0.28	0.22
3	0.27	0.22
4	0.28	0.21
5	0.28	0.21
6	0.27	0.20
7	0.28	0.22
8	0.28	0.21
9	0.28	0.20
10	0.28	0.21
**Mean value**	**0.28**	**0.21**
**Standard deviation**	**0.006**	**0.008**

Supplementary Table 11. Performances of PEMWEs based on reported anode catalysts.

**Anode catalysts**	**Ir loading ($mg\ cm^{-2}$)**	**Current density at 1.9 V ($A\ cm^{-2}$)**	**Stability**	**Degradation rate ($\mu V\ h^{-1}$)**
T-IrO_x-400	0.28	3.0 A	1.793 V@ 2 A cm^{-2} @1600 h	7
IrO_2	0.30	2.4 A	1.920 V@ 2 A cm^{-2} @200 h	230
$IrO_x \cdot nH_2O$[12]	1.6	1.58 A	1.77 V@ 1 A cm^{-2} @600 h	20
Ir-Sn PSC[13]	1.0	1.8 A	1.71 V@ 1 A cm^{-2} @96 h	88.2
IrO_x/Zr_2ON_2[14]	0.4	2 A (1.927 V)	1.71 V@ 1 A cm^2 @50 h	-
Ir-3 MA[15]	0.50	2 A (1.79 V)	1.59 V@ 2 A cm^{-2} @400 h	37
IrO_2@TaB_2[16]	0.15	3.06 A (2.0 V)	1.70 V@ 1 A cm^{-2} @100 h	116.5
Ir-MEO[17]	1	1.43 A (1.7 V)	2.0 V@ 0.5 A cm^{-2} @200 h	629
Ir-ND/ATO[18]	1.4	1.9 A	-	-
IrO_2@TiN_{1+x}[19]	1.6	1.95 A	1.68 V@ 1 A cm^{-2} @100 h	93
Ir_x@$Au_{0.25}Ir_{0.75-x}$[20]	-	1.75 A	1.67 V@ 0.5 A cm^{-2} @100 h	400

Supplementary Table 12. The Gibbs free energy corrections for gas molecules.

	E_{DFT} (eV)	TS (eV)	ZPE (eV)	G (eV)
H_2O	-14.217	0.670	0.582	-14.306
H_2	-6.770	0.410	0.281	-6.899
O_2	\	\	\	-9.894

References

1. Moss, G. C., et al. Perchlorate Fusion–Hydrothermal Synthesis of Nano-Crystalline IrO_2: Leveraging Stability and Oxygen Evolution Activity. *Small* **21**, 2412237 (2025).
2. Yang, L., et al. Efficient oxygen evolution electrocatalysis in acid by a perovskite with face-sharing IrO_6 octahedral dimers. *Nat. Commun.* **9**, 5236 (2018).
3. Lebedev, D., et al. Highly Active and Stable Iridium Pyrochlores for Oxygen Evolution Reaction. *Chem. Mater.* **29**, 5182-5191 (2017).
4. Sun, W., et al. A promising engineering strategy for water electro-oxidation iridate catalysts via coordination distortion. *Chem. Commun.* **55**, 5801-5804 (2019).
5. Kumari, S., Ajayi, B. P., Kumar, B., Jasinski, J. B., Sunkara, M. K. & Spurgeon, J. M. A low-noble-metal $W_{1-x}Ir_xO_{3-\delta}$ water oxidation electrocatalyst for acidic media via rapid plasma synthesis. *Energy Environ. Sci.* **10**, 2432-2440 (2017).
6. Diaz-Morales, O., et al. Iridium-based double perovskites for efficient water oxidation in acid media. *Nat. Commun.* **7**, 12363 (2016).
7. Gao, J., et al. Breaking Long-Range Order in Iridium Oxide by Alkali Ion for Efficient Water Oxidation. *J. Am. Chem. Soc.* **141**, 3014-3023 (2019).
8. Grimaud, A., et al. Activation of surface oxygen sites on an iridium-based model catalyst for the oxygen evolution reaction. *Nat. Energy* **2**, 16189 (2016).
9. Shang, C., et al. Electron Correlations Engineer Catalytic Activity of Pyrochlore Iridates for Acidic Water Oxidation. *Adv. Mater.* **31**, 1805104 (2019).
10. Liu, Z., et al. Electrochemical Preparation of Iridium Hydroxide Nanosheets with Ordered Honeycomb Structures for the Oxygen Evolution Reaction in Acid. *ACS Appl. Energy Mater.* **5**, 6869-6877 (2022).
11. Strickler, A. L., Higgins, D. & Jaramillo, T. F. Crystalline Strontium Iridate Particle Catalysts for Enhanced Oxygen Evolution in Acid. *ACS Appl. Energy Mater.* **2**, 5490-5498 (2019).
12. Xu, J., et al. $IrO_x \cdot nH_2O$ with lattice water–assisted oxygen exchange for high-performance proton exchange membrane water electrolyzers. *Sci. Adv.* **9**, eadh1718 (2023).
13. Zheng, X., et al. Ir-Sn pair-site triggers key oxygen radical intermediate for efficient acidic water oxidation. *Sci. Adv.* **9**, eadi8025.
14. Lee, C., et al. Catalyst-Support Interactions in Zr_2ON_2-Supported IrO_x Electrocatalysts to Break the Trade-Off Relationship Between the Activity and Stability in the Acidic Oxygen Evolution Reaction. *Adv. Funct. Mater.* **33**, 2301557 (2023).
15. Liang, J., et al. Constructing Highly Porous Low Iridium Anode Catalysts Via Dealloying for Proton Exchange Membrane Water Electrolyzers. *Adv. Mater.*, https://doi.org/10.1002/adma.202409386, 2409386 (2024).
16. Wang, Y., et al. Nano-metal diborides-supported anode catalyst with strongly coupled TaO_x/IrO_2

catalytic layer for low-iridium-loading proton exchange membrane electrolyzer. *Nat. Commun.* **14**, 5119 (2023).

17. Yao, L., et al. Sub 2 nm IrRuNiMoCo High-Entropy Alloy with Iridium-Rich Medium-Entropy Oxide Shell to Boost Acidic Oxygen Evolution. *Adv. Mater.* **36**, 2314049 (2024).
18. Oh, H.-S., Nong, H. N., Reier, T., Gliech, M. & Strasser, P. Oxide-supported Ir nanodendrites with high activity and durability for the oxygen evolution reaction in acid PEM water electrolyzers. *Chem. Sci.* **6**, 3321-3328 (2015).
19. Wang, S., et al. Defects tailoring IrO_2@TiN_{1+x} nano-heterojunctions for superior water oxidation activity and stability. *Mater. Chem. Front.* **5**, 8047-8055 (2021).
20. Huang, H., et al. Modulation in the electronic structure of Ir-rich shell on AuIr solid solution as OER electrocatalyst for PEM electrolyzer. *J. Appl. Electrochem.* **54**, 2269-2279 (2024).
