# Supplementary material for: Tunnel-structured IrOx unlocks catalytic efficiency in proton exchange membrane water electrolyzers
Source: Nat Commun. 2025 Aug 15;16:7608. doi: 10.1038/s41467-025-62861-0 (PMC12356887; doi:10.1038/s41467-025-62861-0)
Supplement: Supplementary file 2 — Description of Additional Supplementary Files [file 41467_2025_62861_MOESM2_ESM.pdf]

Description of Additional Supplementary Files

Supplementary Movie 1. The high-speed and microscale visualization system video of R-IrOx.

Supplementary Movie 2. The high-speed and microscale visualization system video of T-IrOx.

Supplementary Data 1. Supplementary Data 1 contains all structures used in the electronic structure calculations described in the manuscript. Atomic coordinates and lattice parameters are provided in plain, unformatted text files (.txt) for maximum compatibility. All structures are fully relaxed. File names reflect tunnel type, surface type, and adsorbate when relevant.
