# Supplementary material for: Tunnel-structured IrOx unlocks catalytic efficiency in proton exchange membrane water electrolyzers
Source: Nat Commun. 2025 Aug 15;16:7608. doi: 10.1038/s41467-025-62861-0 (PMC12356887; doi:10.1038/s41467-025-62861-0)
Supplement: Supplementary file 3 — Supplementary Data 1 [file 41467_2025_62861_MOESM3_ESM.zip › Supplementary_Data_1/Supplementary_Data_1_legend.docx]

**Supplementary_Data_1** contains all structures used in the electronic structure calculations described in the manuscript. Atomic coordinates and lattice parameters are provided in plain, unformatted text files (.txt) for maximum compatibility. All structures are fully relaxed. File names reflect tunnel type, surface type, and adsorbate when relevant.

The folder is organized as follows:

│

**├── bulk**	# All bulk structures

**│	├── no_water_bulk**	# Bulk structures without water, including before & after H+/A+ exchange

**│	└── inner_water_bulk**	# Bulk structures with water inserted into the tunnel

│

**├── slab**	# All slab models

**│	├── surface_energy**	# Slabs for surface energy calculations

**│	├── pourbaix**	# Slabs with various surface coverages for Pourbaix analysis

**│	└── activity**	# Slabs with OER intermediates

**│	├── AEM**	# AEM pathway

**│	├── LOM**	# LOM pathway

**│	└── inner_water_slab**	# AEM-pathway with water inside the tunnel
